# Single-cell transcriptomic atlas of the human testis across the reproductive lifespan

**DOI:** 10.1038/s43587-025-00824-2

**Published:** 2025-03-03

**Authors:** Lina Cui, Xichen Nie, Yixuan Guo, Pengcheng Ren, Yifei Guo, Xiaoyan Wang, Ran Li, James M. Hotaling, Bradley R. Cairns, Jingtao Guo

**Affiliations:** 1 State Key Laboratory of Organ Regeneration and Reconstruction, State Key Laboratory of Stem Cell and Reproductive Biology, Institute of Zoology, Chinese Academy of Sciences, Beijing, China; 2 Beijing Institute for Stem Cell and Regenerative Medicine, Beijing, China; 3 University of the Chinese Academy of Sciences, Beijing, China; 4 Howard Hughes Medical Institute, Department of Oncological Sciences and Huntsman Cancer Institute, Spencer Fox Eccles School of Medicine, University of Utah, Salt Lake City, UT USA; 5 Division of Urology, Department of Surgery, Spencer Fox Eccles School of Medicine, University of Utah, Salt Lake City, UT USA

**Keywords:** Ageing, Transcriptomics

## Abstract

Testicular aging is associated with declining reproductive health, but the molecular mechanisms are unclear. Here we generate a dataset of 214,369 single-cell transcriptomes from testicular cells of 35 individuals aged 21–69, offering a resource for studying testicular aging and physiology. Machine learning analysis reveals a stronger aging response in somatic cells compared to germ cells. Two waves of aging-related changes are identified: the first in peritubular cells of donors in their 30s, marked by increased basement membrane thickness, indicating a priming state for aging. In their 50s, testicular cells exhibit functional changes, including altered steroid metabolism in Leydig cells and immune responses in macrophages. Further analyses reveal the impact of body mass index on spermatogenic capacity as age progresses, particularly after age 45. Altogether, our findings illuminate molecular alterations during testis aging and their relationship with body mass index, providing a foundation for future research and offering potential diagnostic markers and therapeutic targets.

## Abstract (web summary)

To enhance our understanding of aging in the human testis, the authors provide a single-cell map of testicular characteristics throughout the reproductive lifespan. Using samples from 35 donors, they identify two waves of molecular changes in somatic cells and examine interactions between aging and body mass index.

## Main

Female reproductive aging is linked to prolonged time to conception and higher rates of miscarriage^1–4^. While the consequences of male reproductive aging have historically been underestimated, mainly because of ongoing sperm production throughout life^5^, the impact of aging on male fertility is increasingly recognized. This is influenced by several risk factors, including environmental^6–8^ and physiological factors^9,10^.

Aging is a major determinant of diminished male fertility, manifesting through various mechanisms, including cellular dysfunction, heightened disease susceptibility and alterations in reproductive endocrine function. Notably, male reproductive aging can induce conditions resembling some features of the menopause or late-onset hypogonadism^11,12^, which are associated with reduced fertility because of both physiological and molecular changes^13^. Advanced paternal age affects several aspects of sperm and seminiferous tubule function, including serum testosterone levels^14,15^, basement membrane integrity^9,13,16^ and testicular size and weight^13,17^. Moreover, aging affects sperm genome integrity, including telomere length^5,18^, DNA stability^5,19^ and DNA mutations^20^. Changes in germline DNA methylation during aging may contribute to increased offspring disorders^21^. Moreover, aging testes display several changes, such as elevated inflammatory cytokines in testicular somatic cells, metabolic pathway dysregulation in Sertoli cells and Hedgehog signaling pathway dysregulation in Leydig cells, resulting in decreased testosterone production, abnormal proliferation of testicular peritubular cells (TPCs) and decreased spermatogonial stem cell (SSC) counts^9^. Previous research demonstrated that Sertoli cells, in cases of late-onset hypogonadism, exhibit signs of aging and altered lysosomal acidity, which is indicative of disrupted metabolic pathways associated with aging^22^. Plasma proteome analysis across the lifespan identified three peaks of aging-related proteins, especially dynamic extracellular matrix (ECM)-related proteins in humans^23^.

Despite histological studies noting aging-associated testicular changes in humans, nonhuman primates, mice and *Drosophila*, uncertainty remains about how human testicular cell types respond to aging as individuals age. Extensive multi-omics studies, including sperm genomic analyses^24^, testicular transcriptome^9^, seminal plasma proteomics or sperm metabolomics^25,26^ and sperm epigenomics^27,28^, have been conducted to investigate the effects of aging and lifestyle on male fertility. These studies aim to elucidate the impact of aging or lifestyle factors on male fertility. However, despite these efforts, substantial knowledge gaps persist in comprehensively understanding the impact of aging and lifestyle choices on male reproductive health. Heterogeneity among humans and lifestyle factors, such as body mass index (BMI), complicate our understanding of how human testicular clusters respond to aging at the molecular and cellular levels. Therefore, a sufficiently large sample size and well-annotated, high-quality testicular clusters are crucial for gaining systematic and mechanistic insights. Furthermore, our previous work focused on the impact of aging on different testicular cell types from donors at the relative extremes of age, specifically 20-year-olds and 60-year-olds. This initial investigation aimed to determine whether molecular changes occur between younger and older testicular tissue. In the current work, we advance the study scope by collecting a larger number of testicular samples across a broader age range. Our goal is to identify specific time points in the testicular aging response and to understand how several testicular cell types react to aging, along with the underlying molecular mechanisms driving these changes.

Spermatogenesis is a complex process occurring in the seminiferous tubules of the human testis and involves three phases: (1) mitotic division of spermatogonia; (2) meiotic division of spermatocytes; and (3) transformation of round spermatids into elongated spermatids^29–34^. Somatic cells such as Sertoli cells, Leydig cells, TPCs, macrophages and endothelial cells actively participate and regulate this process^30,35,36^. Sertoli cells provide growth factors and nutrients for germ cell development, while Leydig cells synthesize androgens, which are transported to Sertoli cells by androgen-binding proteins^37–44^. TPCs aid intratesticular sperm transport via peristaltic contractions^30,45^. Somatic cells in the testicular microenvironment regulate SSC self-renewal and differentiation through secreted factors^46–49^. Age-related alterations occur in the germline and somatic cell-formed microenvironment. Our investigation of testicular cluster response to aging used single-cell RNA sequencing (scRNA-seq) to characterize individual cells, identify signatures and pathways, delineate germ cell fate determination and address aging-related questions regarding molecular and cellular mechanisms across several age groups^50–55^.

In this study, we constructed a thorough single-cell transcriptomic map of testicular aging using samples from 35 donors aged 21–69, spanning the male reproductive lifespan. This dataset provides us with a valuable opportunity to systematically explore some of the most interesting questions in the field of reproduction, such as whether the aging-related impacts on male subfertility is cumulative or instantaneous and whether different testicular cells respond to aging in a synchronous or asynchronous manner. If the latter, what cell types display the highest sensitivity to aging and what could be the impact? Also, how do aging and obesity function synergistically to impact male fertility? Overall, our research allows us to study the cellular and molecular changes linked to aging in the human testis, identify potential diagnostic markers for testicular aging, demonstrate the efficacy of machine learning in aging studies and furnish a comprehensive dataset covering human testicular profiles from youth to advanced age.

## Results

### Single-cell transcriptomes of human testes at different ages

**Fig. 1:**
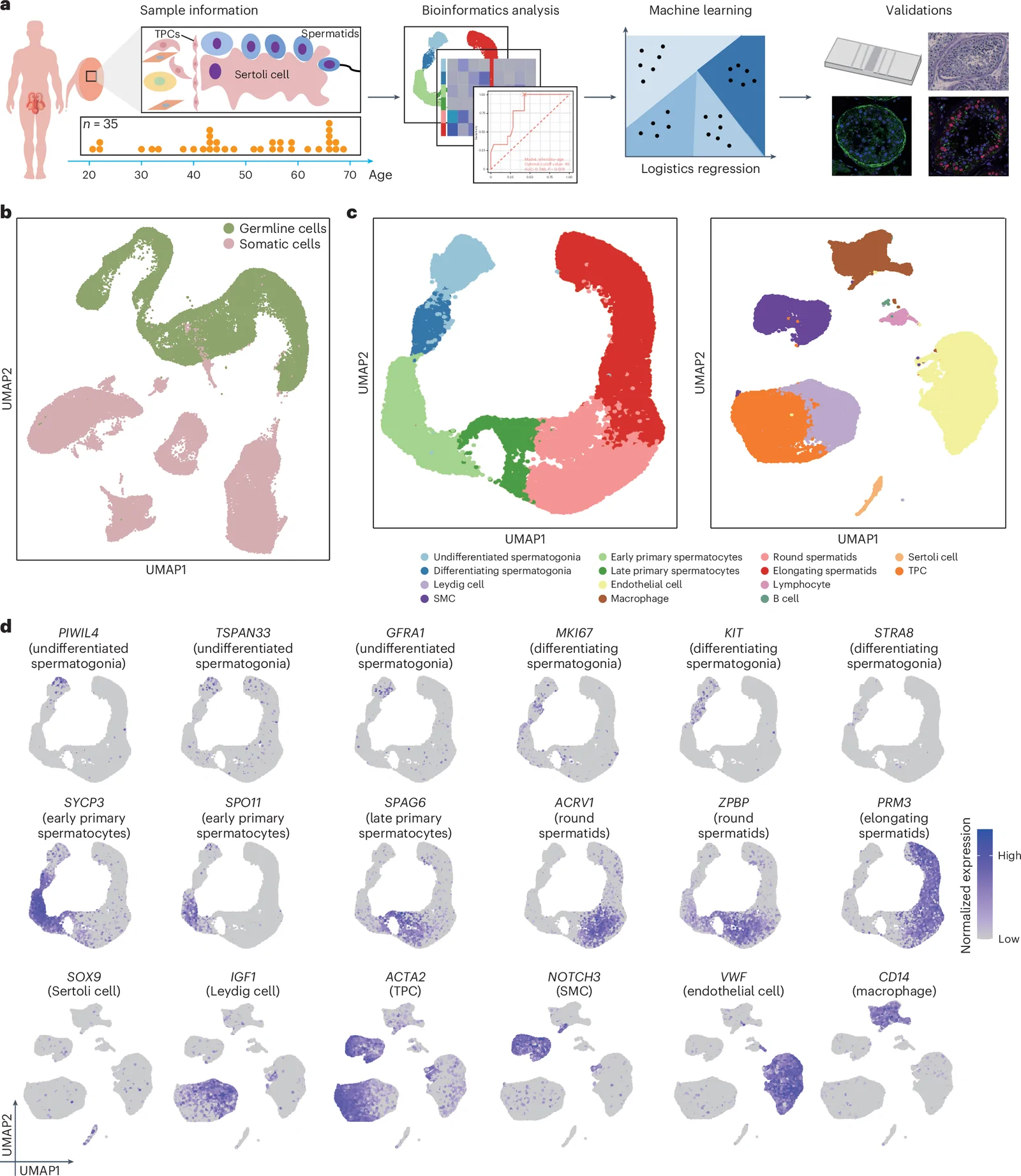
Single-cell transcriptomes of the human testis. **a**, Experimental workflow depicted as a schematic diagram. **b**, UMAP of somatic cells and germ cells in the testes from heathy donors (*n* = 35). Sample sizes: 20s, *n* = 3; 30s, *n* = 4; 40s, *n* = 10; 50s, *n* = 8; 60s, *n* = 10. **c**, UMAP plot with germ (left) and somatic (right) cells from **b** based on the scRNA-seq data from 35 testes. Sample sizes: 20s, *n* = 3; 30s, *n* = 4; 40s, *n* = 10; 50s, *n* = 8; 60s, *n* = 10. **d**, Normalized expression of selected markers identifying major testicular cell types on the UMAP plot. Purple (or gray) represents a high (or low) expression level. Sample sizes: 20s, *n* = 3; 30s, *n* = 4; 40s, *n* = 10; 50s, *n* = 8; 60s, *n* = 10. Source data

**Extended Data Fig. 1:**
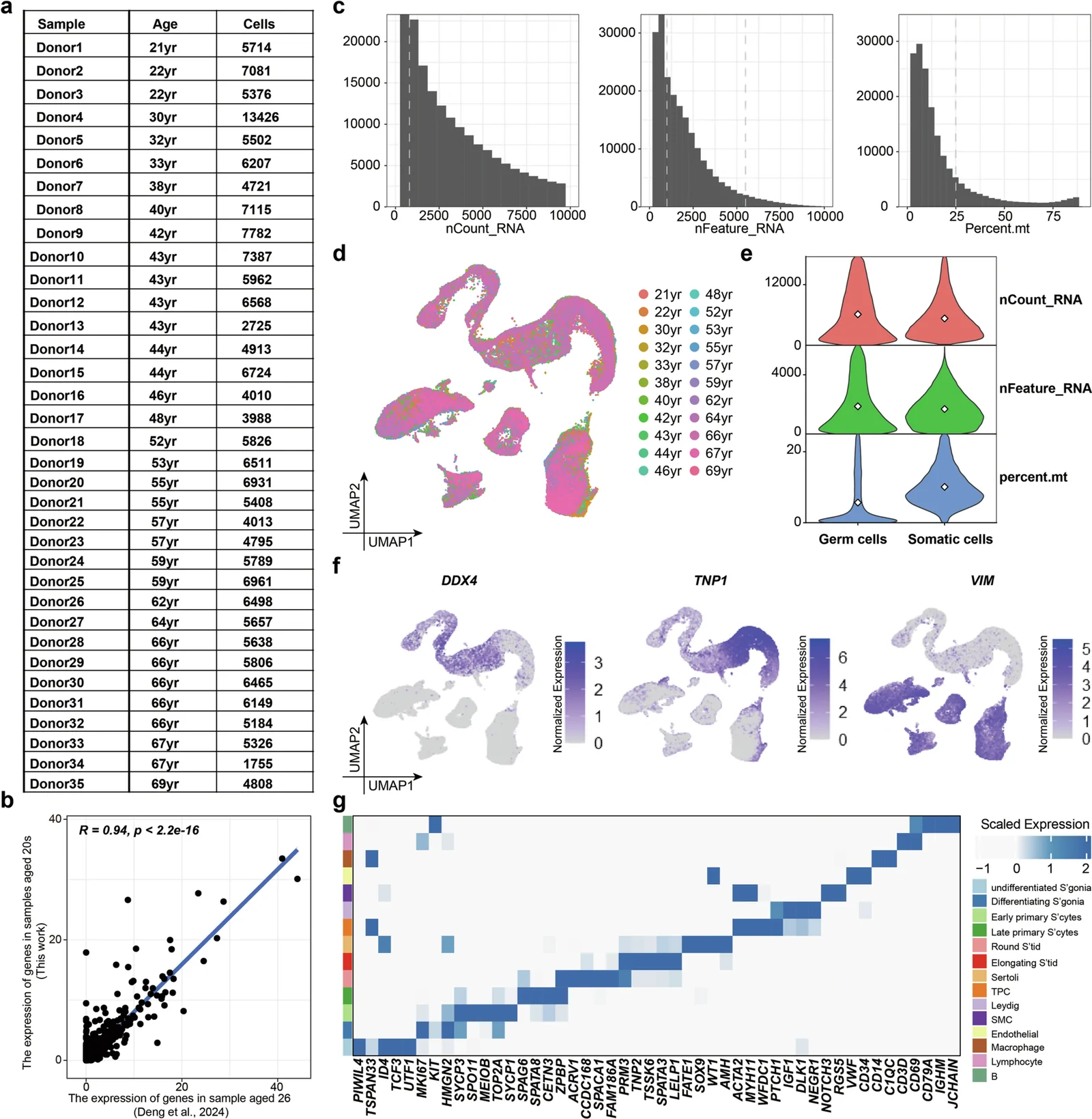
Quality control and clustering. **a**. Information summary of 35 donors in this study. **b**. The pearson correlation coefficient of the samples aged in 20s between our datasets and OA datasets based on highly variable genes. Black dots show the expression level. Permutation test; *P* values presented are unadjusted. **c**. Histogram graph of UMIs/genes/percent.mito. The dashed lines represent the threshold value of quality control. **d**. UMAP plot showing the testicular clusters with color according to the ages of donors. **e**. The stacked violin plots showing the UMIs/genes/percent.mito in somatic cells and germline cells. **f**. UMAP plots showing the normalized expression of *DDX4*, *TNP1*, and *VIM*. **g**. Heatmap with the normalized expression of known and specific marker genes for each cluster. S’gonia, spermatogonia. S’cytes, spermatocytes. S’tids, spermatids. TPC, testicular peritubular cell. SMC, smooth muscle cell. Source data

We gathered 35 human testes from male cadaveric organ donors aged 21–69 years with consistent transcriptional activity (Supplementary Fig. 1). Because the focus of our current study involves the normal physiological process of aging rather than diseased states (for example, infertility), we confirmed that all donors we examined either had offspring or have normal sperm production. Using scRNA-seq on the 10x Genomics Chromium platform, we examined the human testis response to aging, integrating our data with published findings^9^ (Fig. 1a and Extended Data Fig. 1a). Our age range broadly covers the male reproductive lifespan and aligns with previous living testis samples obtained from testicular biopsies of patients with obstructive azoospermia^22^ (Extended Data Fig. 1b). After rigorous quality control, we analyzed 214,369 cells spanning the ages of 21–69 years, with an average of 2,318 genes per cell and an average of 6,265 unique molecular identifiers (UMIs) (Extended Data Fig. 1c). Unsupervised clustering and uniform manifold approximation and projection (UMAP) analysis on the integrated datasets revealed minimal batch effects in each age sample (Extended Data Fig. 1d and Supplementary Fig. 2). Additionally, we identified somatic and germ cells (Fig. 1b and Extended Data Fig. 1e). Based on the expression of *DDX4*, *TNP1* and *VIM* (Extended Data Fig. 1f), we reclustered somatic cells and germ cells into 14 subclusters (Fig. 1c), including undifferentiated spermatogonia (*PIWIL4*, *TSPAN33*), differentiating spermatogonia (*KIT*, *MKI67*), early primary spermatocytes (*SPO11*, *SYCP3*), late primary spermatocytes (*SPAG6*, *SPATA8*), round spermatids (*ACRV1*, *ZPBP*), elongating spermatids (*PRM3*, *TNP2*), Sertoli cells (*SOX9*, *AMH*), TPCs (*ACTA2*, *WFDC1*), Leydig cells (*DLK1*, *IGF1*), smooth muscle cells (SMCs) (*NOTCH3*, *ACTA2*), endothelial cells (*VWF*, *CD34*), macrophages (*C1QC*, *CD14*), lymphocytes (*CD69*, *CD3D*) and B cells (*CD79A*, *IGHM*) (Fig. 1d and Extended Data Fig. 1g). Cell type distribution indicated fertility across all donors (Supplementary Table 1). Thus, we constructed a comprehensive healthy human testis atlas spanning the male reproductive lifespan.

### Cluster specificity in age prediction by aging clocks

**Fig. 2:**
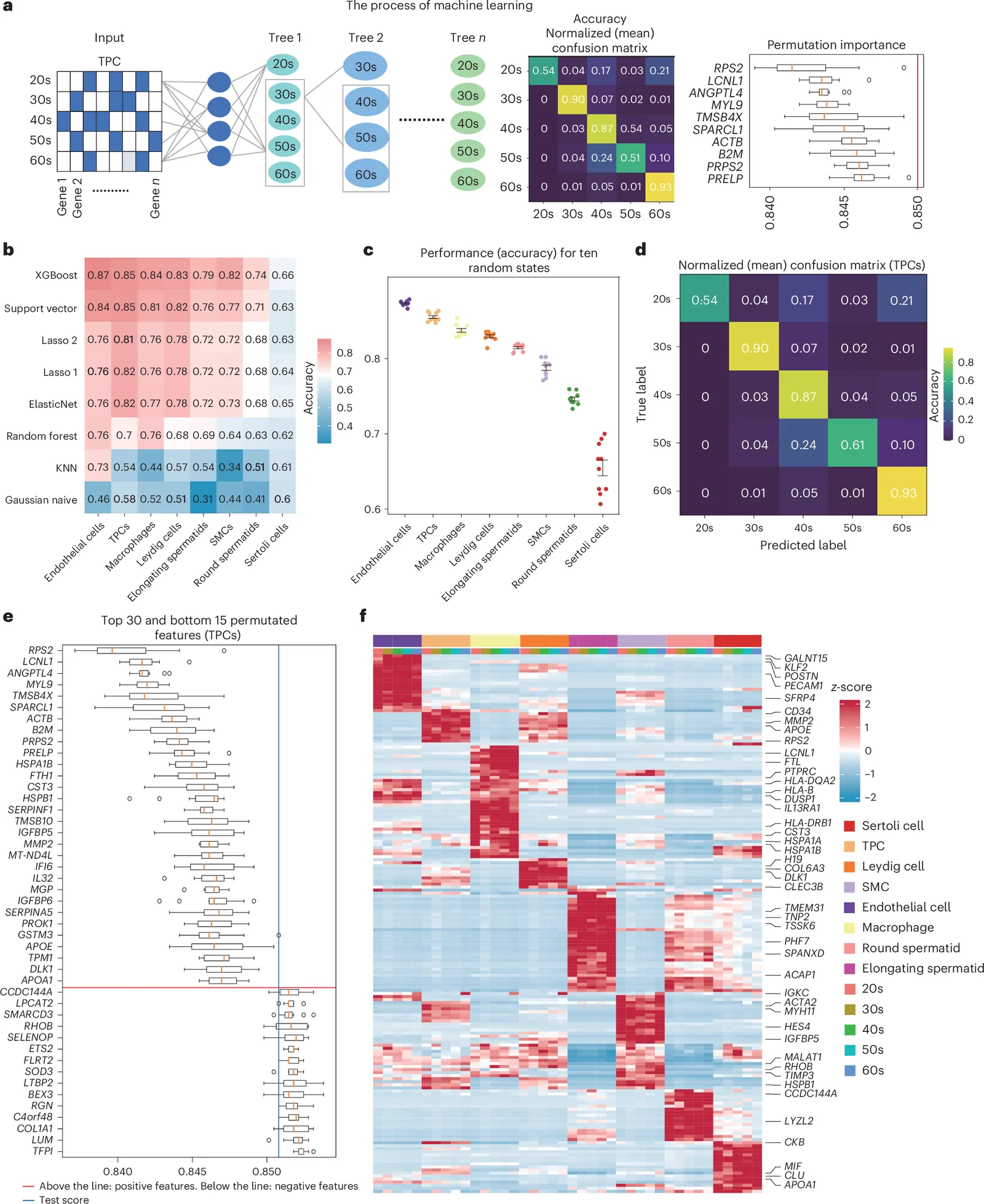
Aging clock analysis shows the presence of cluster specificity in age prediction. **a**, Schematic of the machine learning approach used to predict ages. **b**, Heatmap showing the medium accuracy of eight machine learning models in ten random states. ElasticNet, elastic net regression; KNN, *k*-nearest neighbor classification; Lasso, least absolute shrinkage and selection operator algorithm. Sample sizes: 20s, *n* = 3; 30s, *n* = 4; 40s, *n* = 10; 50s, *n* = 8; 60s, *n* = 10. **c**, Scatter plot showing the accuracy in each testicular cluster of ten random states in the XGBoost model. The bars summarize the s.e. **d**, Heatmap of normalized (mean) confusion matrix in TPCs depicting the accuracy of the XGBoost model. Sample sizes: 20s, *n* = 3; 30s, *n* = 4; 40s, *n* = 10; 50s, *n* = 8; 60s, *n* = 10. **e**, Top 30 and bottom 15 permutated features in TPCs of the XGBoost model. The permutation was repeated ten times. The box plots show the median and the lower and upper quartiles; the whiskers extend to 1.5 times the interquartile range (IQR). The genes above the red line are the ones that influence age prediction. **f**, Heatmap showing the normalized expression of the top 30 genes related to the testicular clusters at different ages based on scRNA-seq data from 35 testes. Right, Representative genes are shown. Sample sizes: 20s, *n* = 3; 30s, *n* = 4; 40s, *n* = 10; 50s, *n* = 8; 60s, *n* = 10. Source data

**Extended Data Fig. 2:**
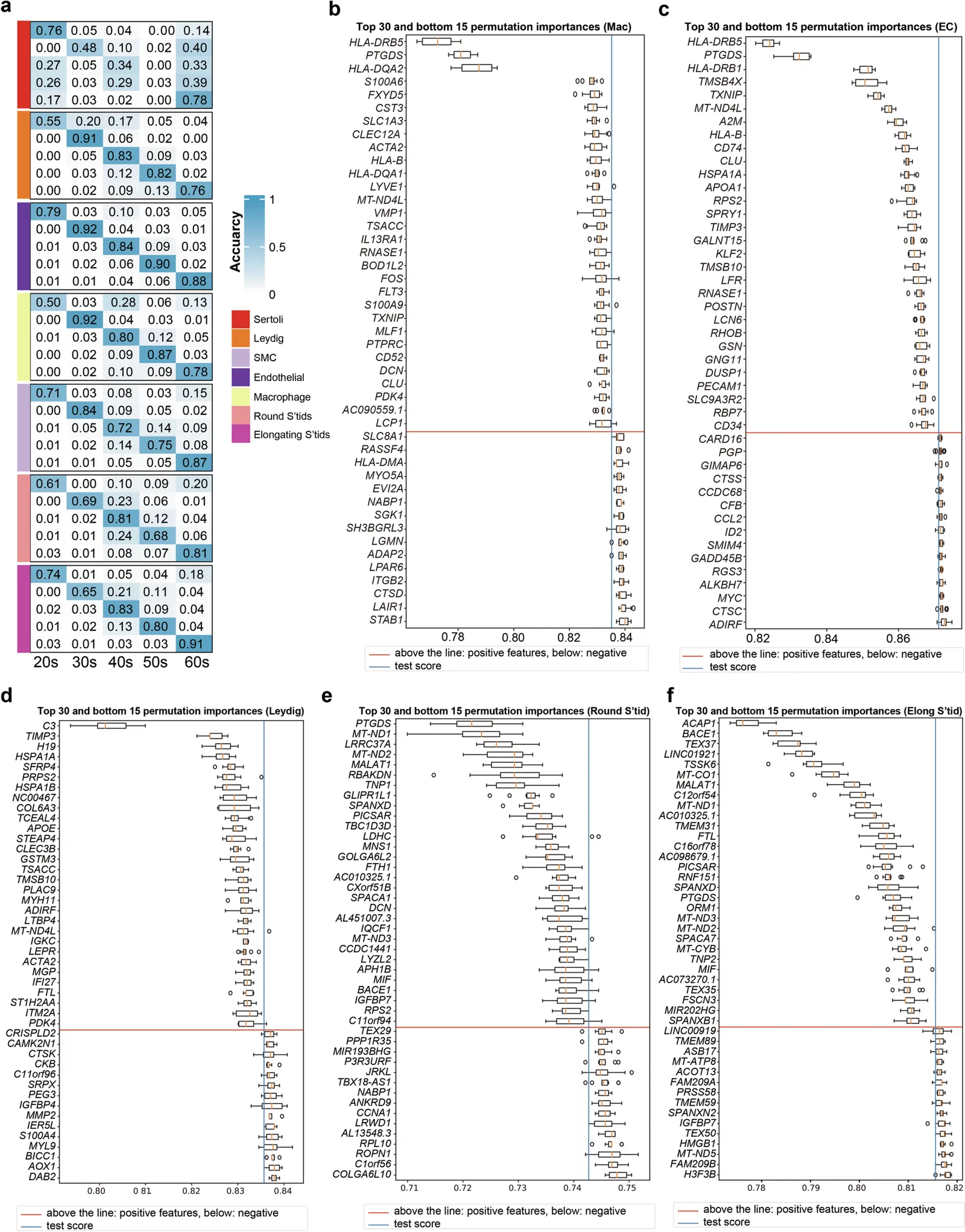
Aging clock analysis. **a**. Heatmap plots showing the accuracy in each cell type based on XGBoost model. S’tids, spermatids. **b–f**. Box plots showing the top 30 and bottom 15 permutated features in macrophage (b), endothelial (c), Leydig (d), round spermatids (e) and elongating spermatid (f). Permutation was repeated ten times. Whisker box plots show median, lower and upper quartiles and whiskers extending to 1.5 times the interquartile range. Source data

We aimed to understand the significance of testicular clusters in predicting biological age. To achieve this, we assessed if the gene expression profiles in these clusters could predict age accurately and uniquely in our dataset (Fig. 2a). Donors were grouped into age brackets spanning their 20s to 60s based on their similarity (Supplementary Fig. 3); we used eight supervised machine learning models for the analysis. Among these models, the extreme gradient boosting classifier (XGBoost)^56^ demonstrated superior performance consistently across all clusters, particularly highlighting the importance of somatic cells in age prediction (Fig. 2b and Supplementary Table 2). Subsequently, we fine-tuned the model to optimize hyperparameters and retrained it using data split across ten random states to address the uncertainties associated with data partitioning and the model’s nondeterministic nature (Fig. 2c). Notably, endothelial cells, TPCs, macrophages, Leydig cells and elongating spermatids exhibited performance exceeding 0.8, underscoring their significance in age prediction. The confusion matrix further validated the model’s performance across each cluster (Fig. 2d and Extended Data Fig. 2a).

To interpret the predictions, we ranked features based on shuffled expression and the importance of the permutation features (Fig. 2e, Extended Data Fig. 2b–f and Supplementary Table 3). Interestingly, we found immune response-related genes in different testicular somatic cell types, suggesting that these genes may impact the accuracy of age prediction in the testicular aging clock and could potentially serve as markers for predicting age-related decline in male fertility. We also examined the corresponding gene expression to investigate potential similarities among key features in each cell type (Supplementary Figs. 4–6). Notably, the expression patterns of the top 30 age-related features in each cluster, influencing age prediction, were cluster-specific (Fig. 2f).

### Consistent and specific changes in somatic cells

**Fig. 3:**
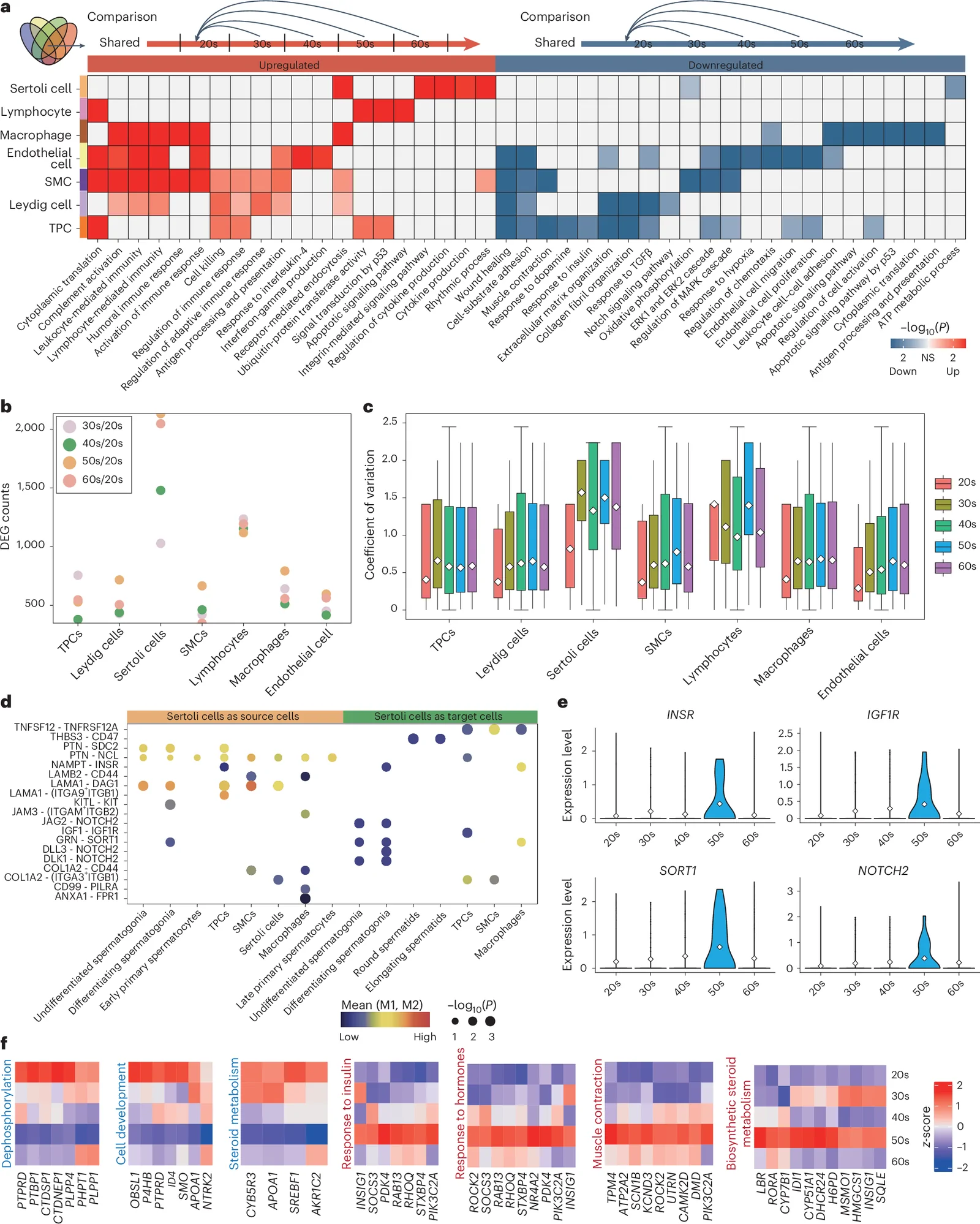
Somatic clusters exhibit a consistent and specific response to the aging process. **a**, Heatmap with the GO terms enriched in the testicular cell types on the basis of shared upregulated and downregulated DEGs compared to individuals in their 20s (two-sided Wilcoxon rank-sum test with multiple comparisons adjusted using the Benjamini–Hochberg method). Sample sizes: 20s, *n* = 3; 30s, *n* = 4; 40s, *n* = 10; 50s, *n* = 8; 60s, *n* = 10. **b**, Scatter plot showing the number of DEGs from different testicular cell types. Each age group is compared with the population in their 20s. Sample sizes: 20s, *n* = 3; 30s, *n* = 4; 40s, *n* = 10; 50s, *n* = 8; 60s, *n* = 10. **c**, Coefficient of variation analysis for each testicular somatic subtype at different ages. In almost all subtypes, the coefficient of variation was significantly changed in individuals in their 30s or 50s (two-sided Wilcoxon rank-sum test with Bonferroni correction). The box indicates the range from the 25th to 75th percentile, with the whiskers extending to 1.5 times the IQR. Outliers are plotted separately. The diamonds indicate the medium value. Sample sizes: 20s, *n* = 3; 30s, *n* = 4; 40s, *n* = 10; 50s, *n* = 8; 60s, *n* = 10. **d**, Overview of ligand–receptor interactions between the Sertoli and testicular clusters at age 50. The means of the average expression level of interacting molecule 1 in Sertoli cells and interacting molecule 2 in testicular clusters are color-coded. Sample sizes: 20s, *n* = 3; 30s, *n* = 4; 40s, *n* = 10; 50s, *n* = 8; 60s, *n* = 10. Permutation test with multiple comparisons adjusted using the Benjamini–Hochberg method. **e**, Violin plot showing the log-normalized expression of *INSR*, *IGF1R*, *SORT1* and *NOTCH2* at different ages in Sertoli cells. The diamonds inside the violin represent the mean. Sample sizes: 20s, *n* = 3; 30s, *n* = 4; 40s, *n* = 10; 50s, *n* = 8; 60s, *n* = 10. **f**, Heatmaps of the scaled expression of significant DEGs in donors in their 50s compared with Leydig cells in donors in their 20s. Left, Enriched pathways (red/blue) indicating upregulation and downregulation. Sample sizes: 20s, *n* = 3; 30s, *n* = 4; 40s, *n* = 10; 50s, *n* = 8; 60s, *n* = 10. Source data

**Extended Data Fig. 3:**
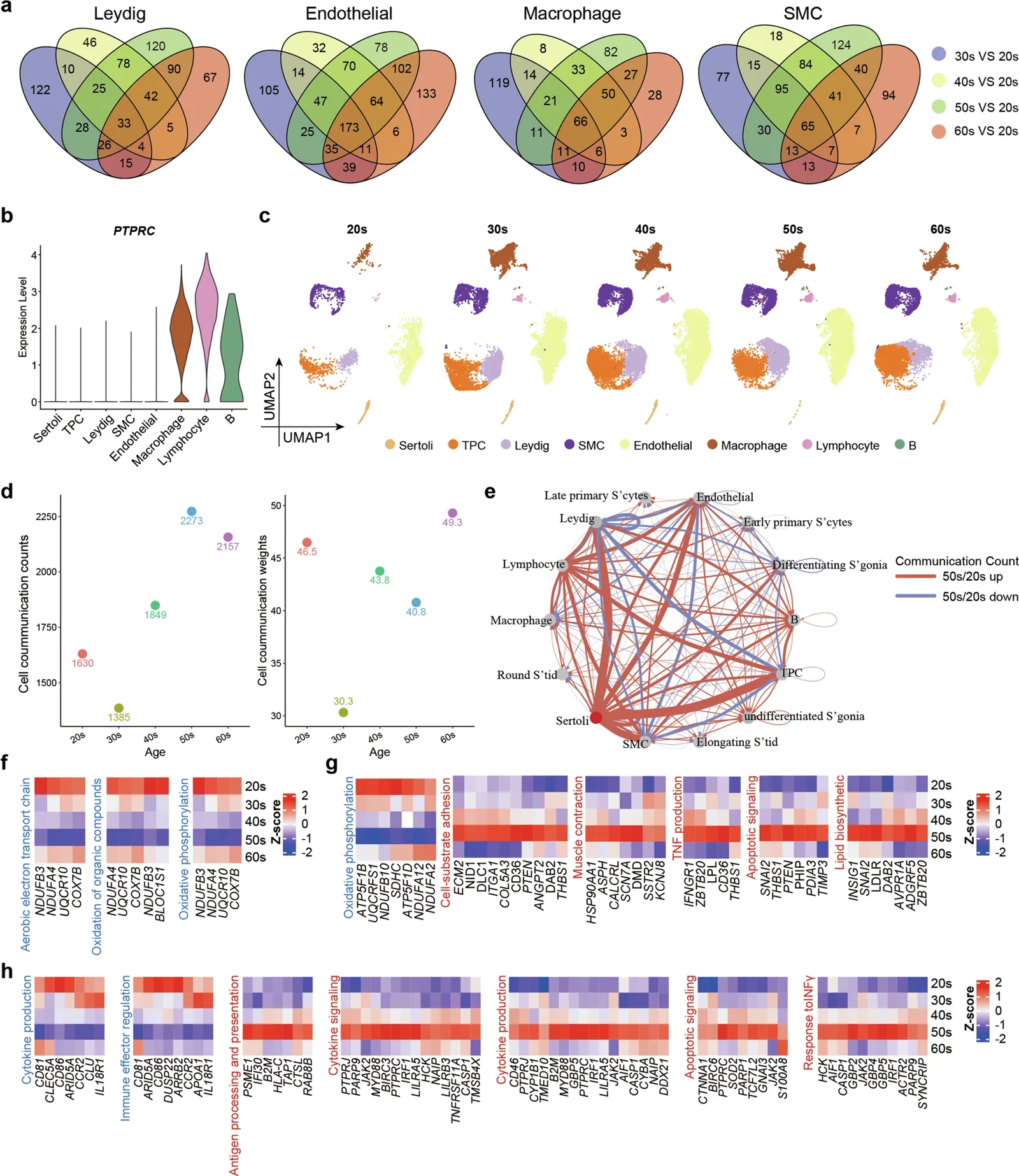
Differentially expressed genes analysis and GO enrichment in somatic cells. **a**. Venn diagrams showing the numbers of significant differentially expressed genes (DEGs) and their overlap in Leydig, endothelial cells, macrophage and smooth muscle cell (SMC). **b**. Violin plot showing the normalized expression of *PTPRC* in somatic cells. TPC, testicular peritubular cell. SMC, smooth muscle cell. **c**. Deconvolution of the UMAP plots according to ages in somatic cells. TPC, testicular peritubular cell. SMC, smooth muscle cell. **d**. Scatter plot showing the cell-cell communication counts (left) and weights (right) in different ages. **e**. Circle plot showing the changed communication counts between 50s and 20s. the lines colored red/blue indicate increase/decrease. **f–h**. Heatmaps of scaled expression of significant DEGs in 50s comparison with 20s in, endothelial cells(f), SMC (g) and macrophage (h). The enriched pathways with bule/red indicate up-regulation/down-regulation were heighted on the left side. SMC, smooth muscle cell. Source data

To understand how supporting somatic cells respond to aging, we conducted a pairwise differential gene expression analysis (Supplementary Fig. 7a), indicating more differentially expressed genes (DEGs) from individuals in their 20s to those from subsequent decades up to their 60s (Supplementary Fig. 7b). This comparison revealed a significant similarity of age-associated changes in gene expression in each somatic cell type, indicating a shared transcriptional response to aging (Extended Data Fig. 3a). We employed Gene Ontology (GO) analysis to identify the significant principal functional pathways affected by aging, finding an enrichment of significantly upregulated DEGs in immune response pathways (Fig. 3a and Extended Data Fig. 3b); significantly downregulated DEGs were predominantly associated with cell-specific pathways, including insulin response, Notch signaling, wound healing, apoptotic processes and the hypoxia response (Fig. 3a). These findings underscore a consistent pattern of gene expression alterations during aging.

Next, a more detailed analysis revealed age-specific DEGs in each somatic cell cluster. Quantifying DEGs across age comparison groups highlighted that TPCs exhibited the highest number of DEGs in samples from donors in their 30s compared to their 20s, whereas Sertoli, Leydig and endothelial cells, and macrophages displayed a marked increase in DEGs in donors in their 50s (Fig. 3b). Coefficient of variation analysis across testicular somatic cell types indicated significant age-related differences, with most cell types exhibiting pronounced changes in individuals in their 50s, except for TPCs, which displayed major changes in individuals in their 30s, potentially contributing to cellular dysfunction later during testis aging (Fig. 3c). Additionally, a reduction in the proportion of Sertoli cells was observed in samples from individuals in their 50s (Extended Data Fig. 3c). Investigations into cellular communication across different ages revealed an escalation in interaction numbers derived from samples from donors in their 50s, particularly in Sertoli cells (Extended Data Fig. 3d,e). Ligand–receptor analysis identified specific ligand–receptor pairs, including NAMPT–INSR, IGF1–IGF1R, PTN–NCL and DLK1–NOTCH2 among others (Fig. 3d). Moreover, receptors such as INSR, IGF1R, SORT1 and NOTCH2 were highly expressed in Sertoli cells from donors in their 50s (Fig. 3e).

To further characterize transcriptomic alterations in Leydig cells, endothelial cells, SMCs and macrophages in response to aging in donors in their 50s, we used differential gene expression and GO analyses to identify pivotal genes and pathways. Leydig cell DEGs constituted 16.8% of all DEGs and were predominantly enriched in pathways related to steroid biosynthesis and metabolism, hormonal and insulin response, aligning with the role of Leydig cells in androgen synthesis, as well as muscle contraction (Fig. 3f). Endothelial cells showed enrichment in oxidative phosphorylation pathways (Extended Data Fig. 3f), while macrophages and SMC DEGs were enriched in tumor necrosis factor cytokine signaling, muscle contraction and ECM organization pathways (Extended Data Fig. 3g,h), respectively.

### TPCs exhibit ECM perturbation in donors in their 30s

**Fig. 4:**
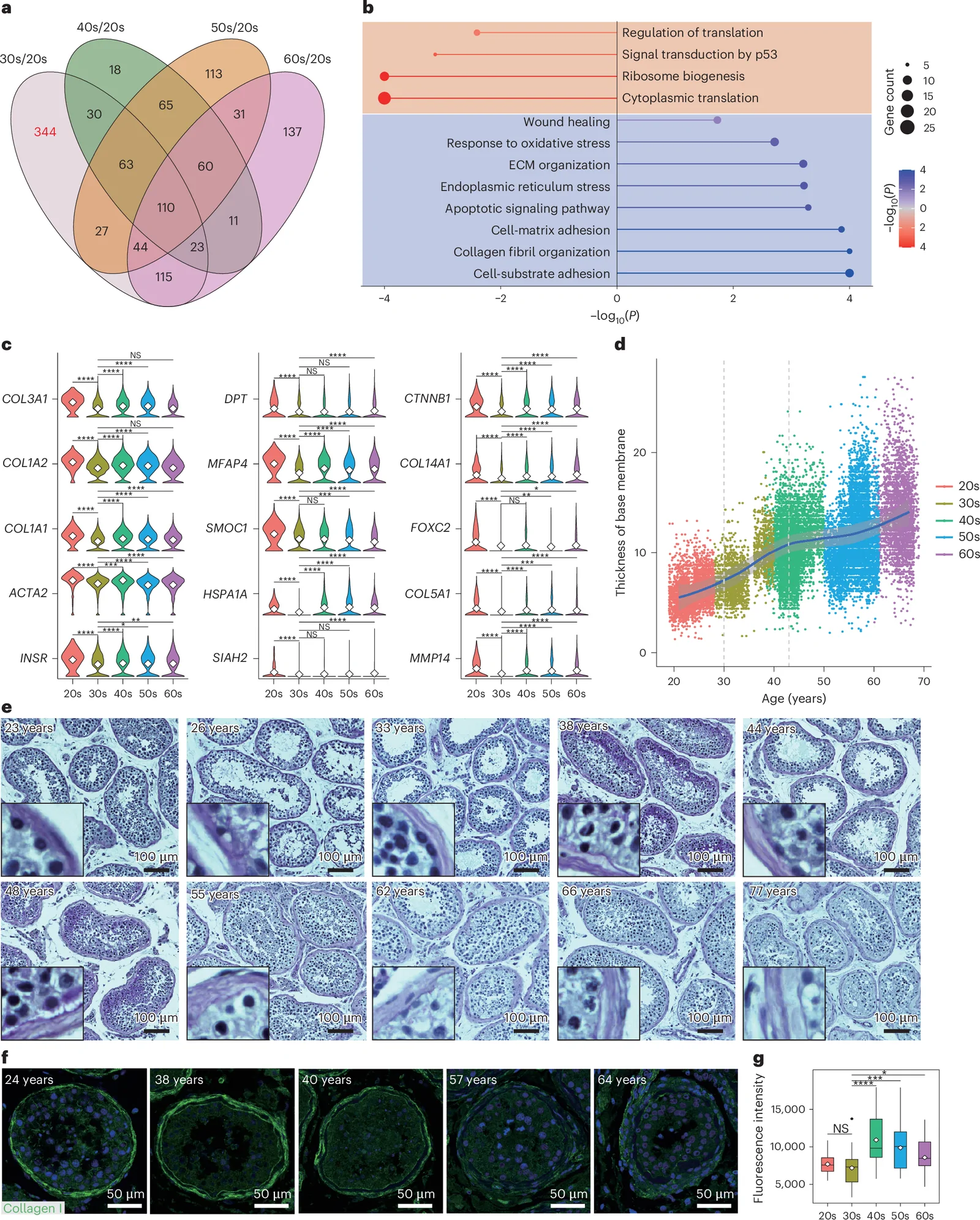
TPCs exhibit rapid and immediate response in ECM and Notch signaling in donors in their 30s. **a**, Venn diagram showing the number of significant DEGs in TPCs and their overlap. Sample sizes: 20s, *n* = 3; 30s, *n* = 4; 40s, *n* = 10; 50s, *n* = 8; 60s, *n* = 10. **b**, Selected GO terms enriched among DEGs specific to donors in their 30s versus donors in their 20s in TPCs. The red and blue dots indicate downregulated and upregulated pathways, respectively. Sample sizes: 20s, *n* = 3; 30s, *n* = 4. **c**, Violin plots showing log-normalized counts of representative genes in TPCs at different ages. The diamond inside the violin represents the mean. Sample sizes: 20s, *n* = 3; 30s, *n* = 4; 40s, *n* = 10; 50s, *n* = 8; 60s, *n* = 10 (two-sided Wilcoxon rank-sum test with Bonferroni correction; ****P ≤ 0.0001; ***P ≤ 0.001; **P ≤ 0.01; *P ≤ 0.05; NS, not significant). *n* = 14,980 iterations on cells from 35 biologically independent samples. **d**, Dot plot with the smoothed line showing the thickness of the basement membrane at different ages from measuring the PAS stain images. The line was smoothed by locally weighted regression (locally estimated scatterplot smoothing). The regression fit to the trend is shown along with the 95% confidence interval bands. The samples include individuals with high and low BMI. Sample sizes: 20s, *n* = 3; 30s, *n* = 4; 40s, *n* = 10; 50s, *n* = 8; 60s, *n* = 10. **e**, PAS staining images of testes at different ages showing a significant increase in staining signal in the basement membrane. Lower left, Images are the localized zoom-in view at the same magnification. Two donors from each group are shown. **f**, IF staining results showing the location of collagen I and 4′,6-diamidino-2-phenylindole (DAPI) in human testes. Two donors from each group are shown. **g**, Box plots showing the mean fluorescence intensity in each tube (*n* = 15). The diamond inside the whiskers represents the mean. The black point outside the whiskers represents outliers. Two-sided *t*-test with Bonferroni correction; ****P = 6.4 × 10^−7^; ***P = 0.00017; *P = 0.01; NS, *P* > 0.05. Two donors from each group are shown. The box plots show the median and lower and upper quartiles; the whiskers extend to 1.5 times the IQR. Each age group includes individuals with high and low BMI. Source data

**Extended Data Fig. 4:**
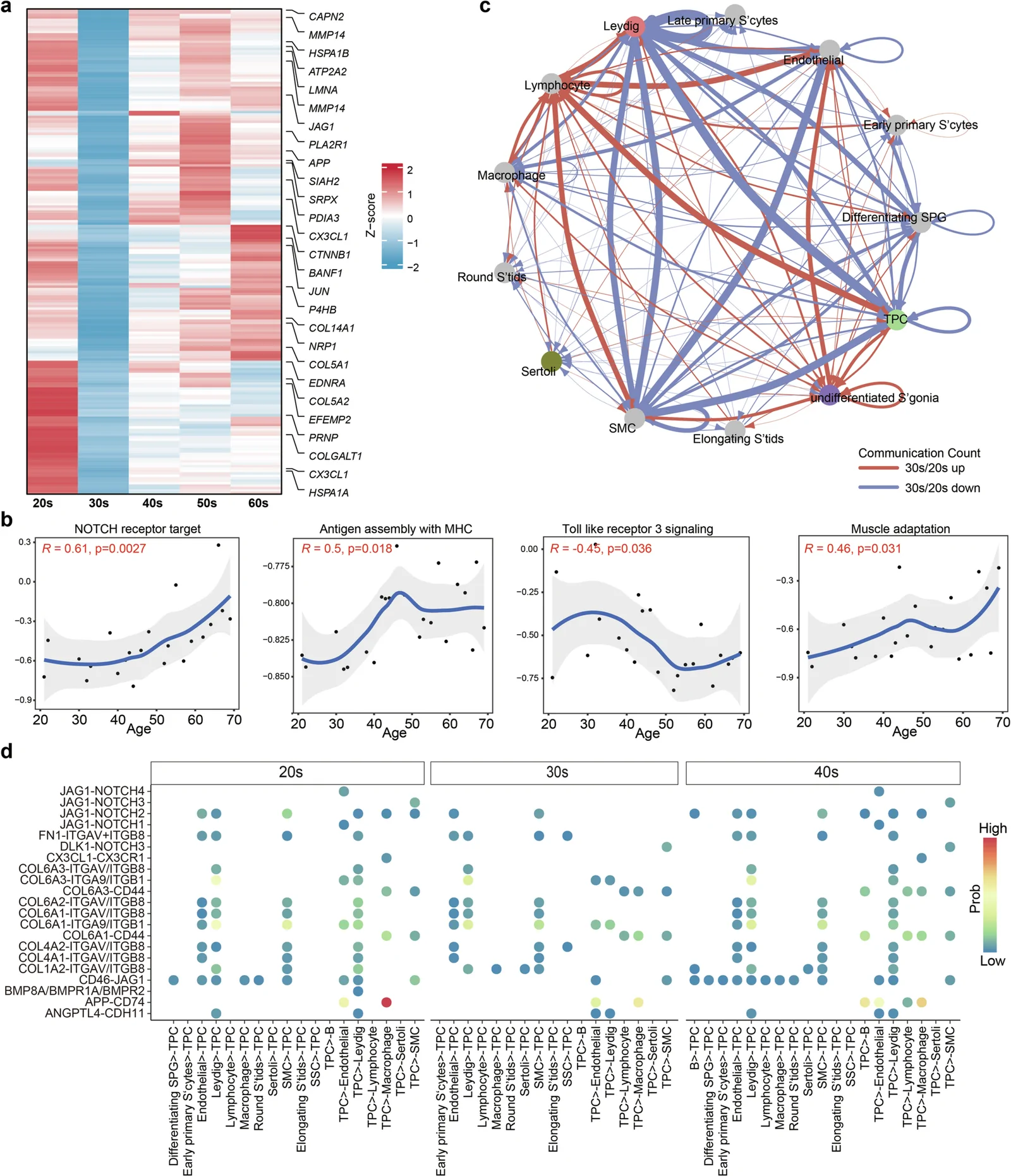
Analysis of aging in testicular somatic cells. **a**. Heatmap showing the expression of differentially expressed genes between 30s versus 20s. **b**. Line plots showing the dynamic expression of NOTCH receptor target, antigen assembly with MHC, Toll-like receptor 3 signaling, and muscle adaptation pathways by GSVA analysis. The loess regression fit to the trend is shown along with the 95% confidence interval bands. Permutation test with multiple comparisons adjusted by the Benjamini–Hochberg method. **c**. Circle plot showing the changed communication counts between 30s and 20s. The lines colored red/blue indicate increase/decrease. **d**. Bubble plot showing the cell-cell communications between TPC and testicular cell types. S’cytes, spermatocytes. S’tids, spermatids. TPC, testicular peritubular cell. SMC, smooth muscle cell. Source data

We revealed that TPCs display very significant aging-correlated changes as early as donors in their 30s. While previous research documented aging responses in TPCs involving the ECM and collagen, the precise nature of this response—whether it occurs gradually or episodically—is to be fully understood. To investigate this specificity, we focused on TPCs. A notable proportion, comprising 26% of all DEGs, underwent distinct alterations in samples from donors in their 30s, indicating the early onset of an age-related response in TPCs (Fig. 4a and Supplementary Fig. 7c). Functional enrichment analysis of these DEGs revealed significant upregulation in pathways related to cytoplasmic translation (for example, *RPL38*, *RPL37*, *RPL17*), p53-mediated signal transduction and ribosome biogenesis (Fig. 4b). Conversely, downregulated pathways were associated with the oxidative stress response (for example, *HSPA1A*, *HSPA1B*, *CTNNB1*), the ECM pathway (for example, *MMP14*, *COL14A1*, *COL5A2*, *COL5A1*, *COLGALT1*, *EFEMP2*, *APP*) and collagen fibril organization (for example, *COL14A1*, *COL5A2*, *COL5A1*, *LMNA*, *SIAH2*) (Fig. 4b). The expression patterns of related genes confirmed an early-onset response involving the ECM pathway in donors in their 30s (Fig. 4c and Extended Data Fig. 4a).

Gene set variation analysis (GSVA) further revealed that pathways such as Notch signaling, Toll-like receptor 3 signaling, antigen processing and presentation, and muscle adaptation exhibited a significant upward trend with advancing age (Extended Data Fig. 4b). To validate these findings, periodic acid–Schiff (PAS) staining was performed on all samples, indicating an age-associated increase in basement membrane thickness in donors during their 30s (Fig. 4d,e and Supplementary Fig. 7d). Moreover, quantitative analysis of collagen I supported substantial changes in TPCs in donors during their 30s (Fig. 4f,g and Supplementary Fig. 7e). To further explore how alterations in TPCs surrounding the seminiferous tubules impact the spermatogenic microenvironment, we conducted CellChat analysis. The results delineated modifications in communication between TPCs and other testicular cell types in donors during their 30s compared to donors in their 20s (Extended Data Fig. 4c,d). Taken together, these findings suggest a decrease in ECM secretion and oxidative stress response, coupled with an increase in cytoplasmic translation, as part of the age-related changes occurring in TPCs in donors during their 30s.

### Haploid germ cells display pronounced changes during aging

**Fig. 5:**
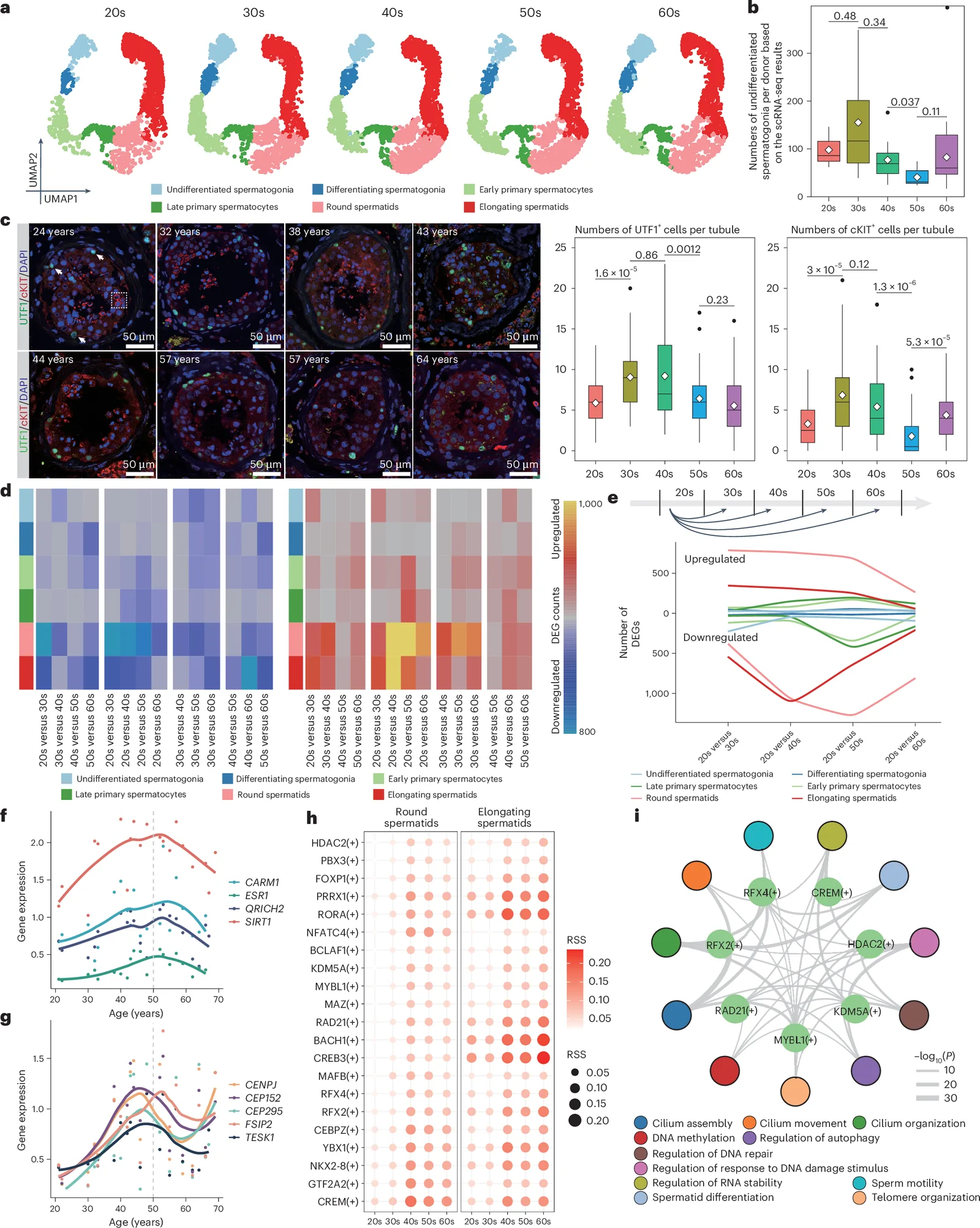
Analysis of aging in spermatogonia and haploid germ cells. **a**, Deconvolution of the UMAP plot according to ages. Sample sizes: 20s, *n* = 3; 30s, *n* = 4; 40s, *n* = 10; 50s, *n* = 8; 60s, *n* = 10. **b**, Box plots showing the numbers of undifferentiated spermatogonia per donor (*n* = 35). Sample sizes: 20s, *n* = 3; 30s, *n* = 4; 40s, *n* = 10; 50s, *n* = 8; 60s, *n* = 10. Two-sided *t*-test with Bonferroni correction; *P* values are shown. Two donors from each group are shown. The box plots show the median and lower and upper quartiles; the whiskers extend to 1.5 times the IQR. The black points outside the whiskers represent outliers. **c**, Left, IF staining showing the localization of UTF1 and cKIT in each donor. The white arrows indicate UTF1^+^ cells and the white dashed square indicates cKIT^+^ cells. Two donors from each group were used to validate the results. Right, Box plots showing UTF1/cKIT^+^ cells per tube (*n* = 25). Two-sided *t*-test with Bonferroni correction; *P* values are shown. The box plots show the median and lower and upper quartiles; the whiskers extend to 1.5 times the IQR. The black points outside the whiskers represent outliers. **d**, Heatmap of the numbers of DEGs in testicular germ cells for pairwise comparisons. Sample sizes: 20s, *n* = 3; 30s, *n* = 4; 40s, *n* = 10; 50s, *n* = 8; 60s, *n* = 10. **e**, Line plot displaying DEGs for pairwise comparisons. Sample sizes: 20s, *n* = 3; 30s, *n* = 4; 40s, *n* = 10; 50s, *n* = 8; 60s, *n* = 10. **f**,**g**, Dot plot with the smoothed line showing the expression of representative genes in round (**f**) and elongating (**g**) spermatids along the ages. Sample sizes: 20s, *n* = 3; 30s, *n* = 4; 40s, *n* = 10; 50s, *n* = 8; 60s, *n* = 10. **h**, Dot plots showing the regulon specificity score (RSS) of representative regulons in round (left) and elongating (right) spermatids. Sample sizes: 20s, *n* = 3; 30s, *n* = 4; 40s, *n* = 10; 50s, *n* = 8; 60s, *n* = 10. **i**, Regulatory network showing the pathways enriched by the targets of representative regulons in donors in their 50s. Permutation test with multiple comparisons adjusted using the Benjamini–Hochberg method. Sample size: 50s, *n* = 8. Source data

**Extended Data Fig. 5:**
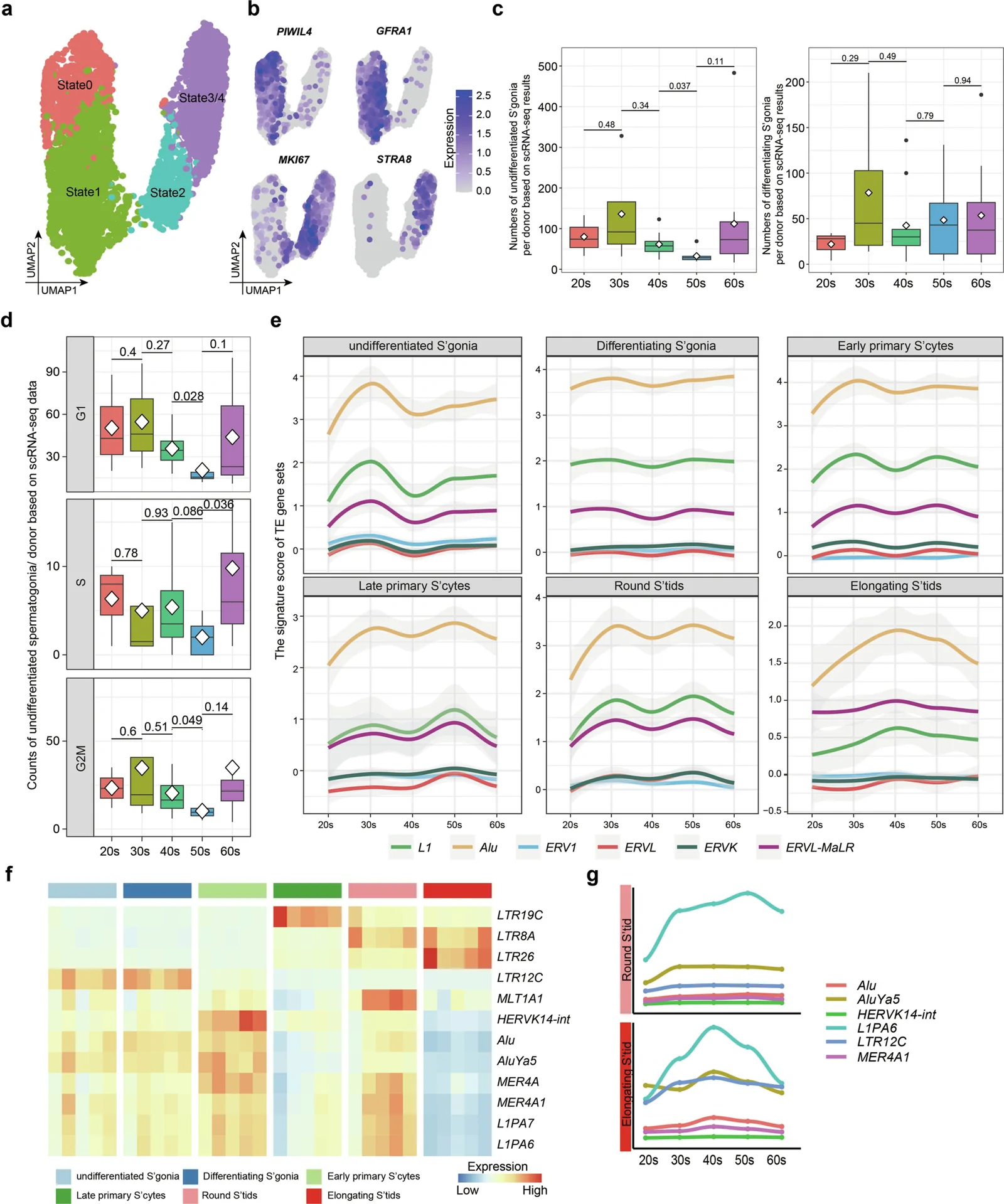
Sub-clustering of spermatogonia and transposable elements analysis. **a**. UMAP plot showing the subtypes of spermatogonia. **b**. UMAP plot showing the normalized expression of *PIWIL4*, *GFRA1*, *MIK67*, and *STRA8*. **c**. The box plots showing the number of undifferentiated spermatogonia (left) and differentiating spermatogonia (right) in each group based on scRNA-seq datasets. Diamonds inside the whiskers represent the mean. Two-sided t-test with Bonferroni correction, *P* values are indicated. Whisker box plots show median, lower and upper quartiles and whiskers extending to 1.5 times the interquartile range. Sample sizes: 20s, *n* = 3; 30s, *n* = 4; 40s, *n* = 10; 50s, *n* = 8; 60s, *n* = 10. **d**. Boxplots showing the numbers of undifferentiated spermatogonia from each age in the phase G1, S or G2/M of the cell cycle, as predicted from their transcriptomic profiles. Diamonds inside the whiskers represent the mean. Two-sided t-test with Bonferroni correction, *P* values are indicated. Whisker box plots show median, lower and upper quartiles and whiskers extending to 1.5 times the interquartile range. Sample sizes: 20s, *n* = 3; 30s, *n* = 4; 40s, *n* = 10; 50s, *n* = 8; 60s, *n* = 10. **e**. Dynamic expression of subtypes of transposable elements in each germline cells. S’gonia, spermatogonia. S’cytes, spermatocytes. S’tids, spermatids. Sample sizes: *n* = 35. The loess regression fit to the trend is shown along with the 95% confidence interval bands. **f**. Heatmap plots showing the normalized expression of *LTR19C*, *LTR8A*, *LTR26*, *LTR12C*, *MLT1A1*, *HERVK14-int*, *Alu*, *AluYa5*, *MER4A*, *MER3A1*, *L1PA7*, and *L1PA6* in germline clusters. S’gonia, spermatogonia. S’cytes, spermatocytes. S’tids, spermatids. **g**. Line plots showing the related transposable elements in round spermatids and elongating spermatids. S’tids, spermatids. Source data

While spermatogenesis is observed across all age groups, certain samples displayed reduced spermatid counts (Fig. 5a). Initial analysis of the number of undifferentiated spermatogonia across different donors revealed a significant decrease with aging (Fig. 5b). Consequently, we referred to prior studies^9^ and reclustered spermatogonia into undifferentiated spermatogonia (state 0 (*PIWIL4*), state 1 (*GFRA1*)) and differentiating spermatogonia (state 2 (*MKI67*), state 3/4 (*STRA8*)) (Extended Data Fig. 5a,b). The number of undifferentiating spermatogonia across age groups revealed a significant decrease in donors during their 50s (Extended Data Fig. 5c and Supplementary Fig. 8a); undifferentiated spermatogonia exhibited significant changes in the G2/M phase (Extended Data Fig. 5d and Supplementary Fig. 8b), suggesting a decline in SSC proliferation. To further validate the decline in the quantity of spermatogonia, we performed immunofluorescence (IF) staining in each group. Quantification and statistical analysis revealed that the number of spermatogonia increased in donors during their 30s and declined in donors during their 50s, indicating significant age-related changes in the quantity of spermatogonia (Fig. 5c).

Subsequently, pairwise differential expression analysis was conducted to determine the emergence of DEGs and their persistence with advancing age (Fig. 5d). While minimal DEGs were observed between clusters at adjacent age groups, most clusters exhibited a significant increase in DEGs in older germline cells compared to those in their 20s, suggesting a progressive and gradual shift in gene expression that is more pronounced with advanced aging stages (Fig. 5e). Certain clusters, such as undifferentiated and differentiating spermatogonia, were relatively resistant to age-related gene expression changes, corroborating previous studies^9^, which suggested minimal transcriptome changes in spermatogonia (Fig. 5e). Notably, DEGs emerged in round and elongating spermatids in donors during their 40s or 50s, indicating a pronounced age-related transformation in the transcriptome of haploid germ cells (Fig. 5e).

Furthermore, transposable elements (TEs), known to become activated and contribute to the aging process^57^, display dynamic expression patterns during mouse spermatogenesis^58^. To explore whether TE expression changes during aging in human spermatogenesis, we used single-cell TE (scTE), a pipeline designed for identifying TEs at the single-cell level^59^ based on BAM files, for TE analysis. Our analyses revealed an age-related increase in specific TEs (including the LINE1, Alu and ERV families), particularly evident in round and elongating spermatids (Extended Data Fig. 5e). Notably, *LTR12C* exhibited high expression in spermatogonia (Extended Data Fig. 5f). Additionally, we observed elevated expression of several TEs, such as *Alu*, *L1PA6* and *MER4A1*, in haploid germ cells from donors aged 40–50 years (Extended Data Fig. 5g).

### Gene expression changes in haploid germ cells during aging

**Extended Data Fig. 6:**
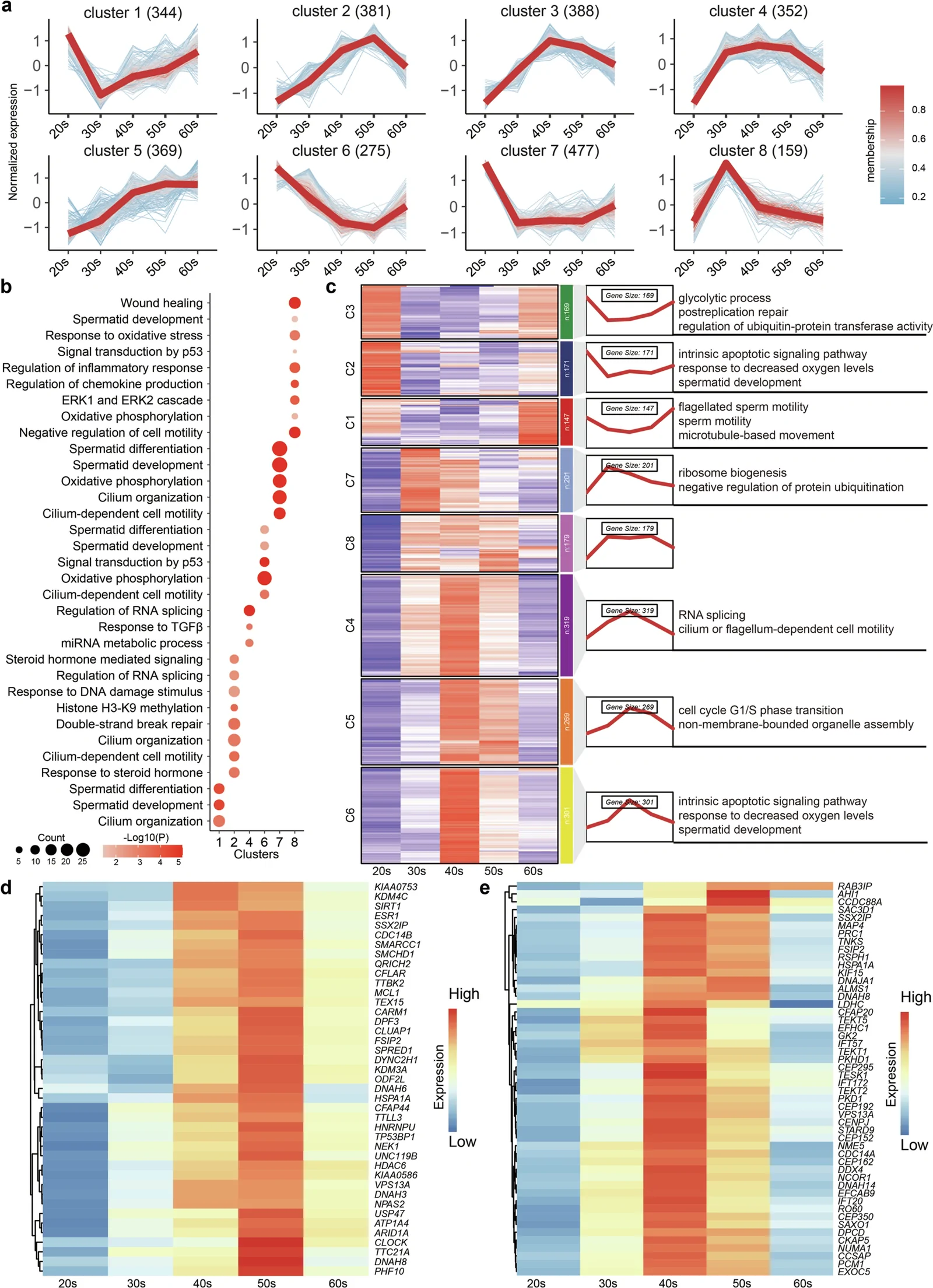
Expression of genes associated with flagella and spermatid morphology underwent largely alterations during aging in haploid germ cells. **a**. Dynamic expression along ages in round spermatids based on fuzzy c-means algorithm. **b**. Bubble plot showing the enrichment of genes from Extended Data Fig. 6a. The hypergeometric *P* values were adjusted for multiple testing using the Benjamini–Hochberg method. **c**. Heatmap and line plots showing the dynamic expression along ages in elongating S’tids. S’tids, spermatids. **d, e**. The heatmap showing the gene expression patterns along the ages in round spermatids (d) and elongating spermatids (e). Source data

To uncover the molecular characteristics underlying haploid germ cell aging at 40–50 years, we examined gene expression across age groups between round and elongating spermatids. By calculating the average expression for significant DEGs collected from pairwise comparisons across ages, we identified significant aging-related pathways, such as cilium-dependent or flagellum-dependent cell motility, spermatid development and double-strand break repair (Extended Data Fig. 6a–c). Interestingly, we observed consistent transcriptional changes in round and elongating spermatids in donors in their 50s. For instance, genes enriched in cluster 5 (Extended Data Fig. 6a) demonstrated a strong increase in expression across ages in round spermatids, while genes in cluster 4 exhibited a sharp increase in elongating spermatids in donors during their 40s and 50s (Extended Data Fig. 6c) relative to other ages. Considering heterogeneity among donors, we presented the expression of significant aging-related functional genes in each donor. For example, significant DEGs enriched on flagellum-dependent functions and spermatid morphology pathways, such as *CARM1* (ref. ^60^), *ESR1* (ref. ^61^), *QRICH2* (ref. ^62^) and *SIRT1* (refs. ^63,64^), which were highly expressed in donors during their 50s in round spermatids, displayed consistent dynamics across each age group (Fig. 5f and Extended Data Fig. 6d). Similarly, DEGs such as *CENPJ*^65^, *FSIP2* (refs. ^66,67^), *TESK1*, *CEP152* (ref. ^68^) and *CEP295* in elongating spermatids also demonstrated dynamic patterns (Fig. 5g and Extended Data Fig. 6e). Additionally, we used SCENIC^69^ to identify key regulons that may regulate gene expression during aging in haploid germ cells. The top ten transcription factors whose regulons were enriched in haploid germ cells exhibited a dynamic pattern across ages, especially in donors in their 50s (Fig. 5h). Subsequent gene regulatory network analysis illustrated that regulons participate in significant functional pathways, such as cilium assembly, regulation of autophagy, sperm motility, spermatid differentiation and regulation of response to DNA damage stimulus (Fig. 5i), supporting the significant age-related changes in haploid germ cells in donors in their 50s.

### Impact of BMI on spermatogenesis increases with advancing age

**Fig. 6:**
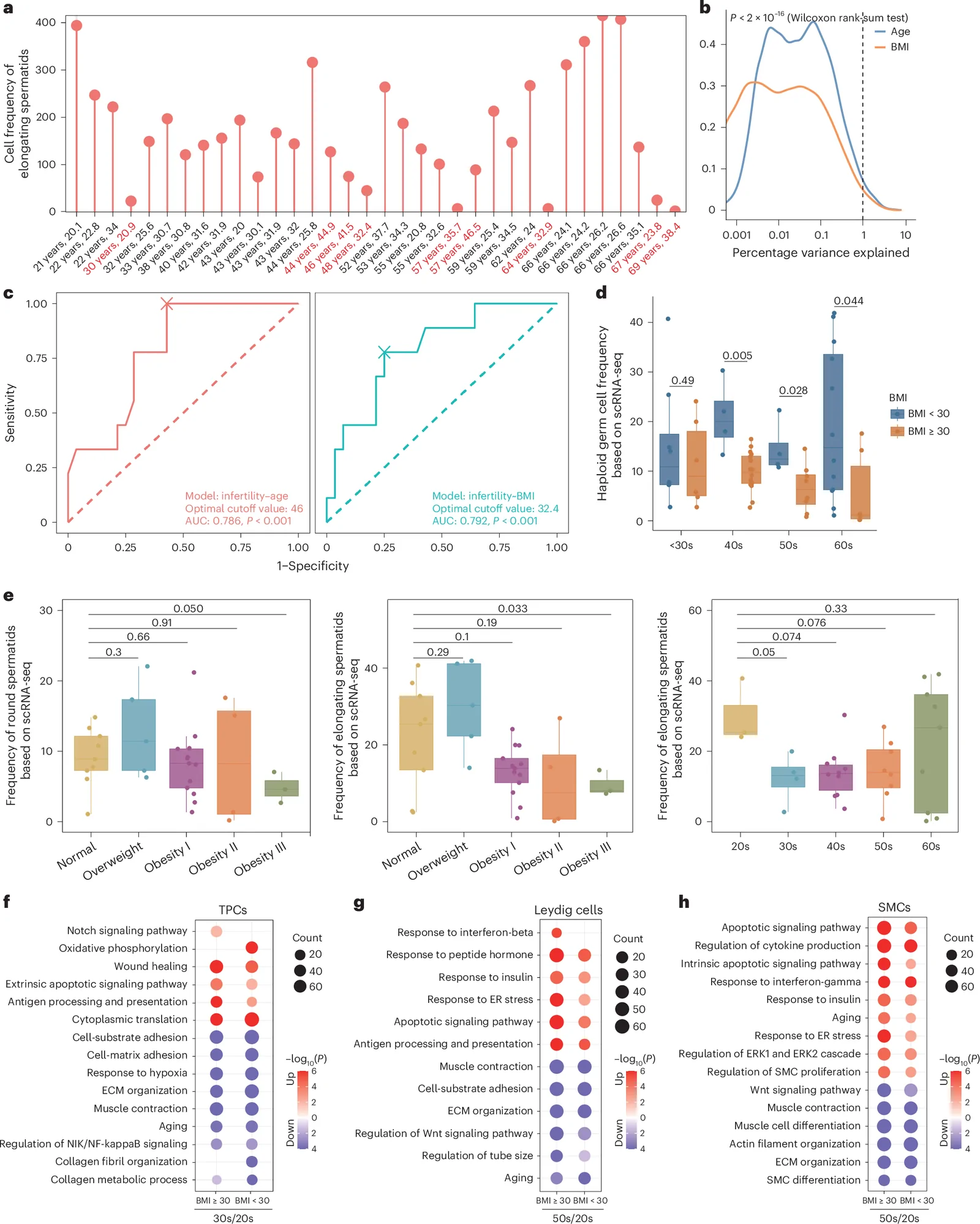
The impact of BMI progressively influences spermatogenic capacity as age advances. **a**, Lollipop chart showing the frequency of elongating spermatids in each donor. The red text indicates that the frequency of elongating spermatids is less than 20. **b**, Density plot showing the percentage of variance explained according to age and BMI across all genes (two-sided Wilcoxon rank-sum test with Bonferroni correction; *P* values are shown). Sample sizes: 20s, *n* = 3; 30s, *n* = 4; 40s, *n* = 10; 50s, *n* = 8; 60s, *n* = 10; BMI ≥ 30, *n* = 21; BMI < 30, *n* = 14. **c**, Receiver operating characteristic (ROC) curve showing the specificity and sensitivity in predicting infertility according to age (left) and BMI (right). Sample sizes: 20s, *n* = 3; 30s, *n* = 4; 40s, *n* = 10; 50s, *n* = 8; 60s, *n* = 10 (two-sided Wilcoxon rank-sum test with Bonferroni correction; *P* values are shown). **d**, Box plot showing the distribution of the frequency of haploid germ cells (round and elongating spermatids) between low (BMI < 30) and high (BMI ≥ 30) BMI at different ages based on the scRNA-seq analysis. Sample sizes: 20s, *n* = 3; 30s, *n* = 4; 40s, *n* = 10; 50s, *n* = 8; 60s, *n* = 10; BMI ≥ 30, *n* = 21; BMI < 30, *n* = 14 (two-sided *t*-test with Bonferroni correction; *P* values are shown). The box plots show the median and lower and upper quartiles; the whiskers extend to 1.5 times the IQR. **e**, Distribution of frequency of round (left) or elongating (middle and right) spermatids according to different BMIs and ages. Sample sizes: normal, BMI < 25, *n* = 9; overweight, 25 < BMI ≤ 30, *n* = 5; obesity I, 30 < BMI ≤ 35, *n* = 14; obesity II, 35 < BMI ≤ 40, *n* = 4; obesity III, BMI > 40, *n* = 3 (two-sided *t*-test with Bonferroni correction; *P* values are shown). The box plots show the median and lower and upper quartiles; the whiskers extend to 1.5 times the IQR. **f**–**h**, Dot plot showing the enrichment in TPCs (**f**), Leydig cells (**g**) and SMCs (**h**). The red and blue dots indicate upregulated and downregulated pathways in BMI ≥ 30 and BMI < 30. Sample sizes: BMI ≥ 30, *n* = 21; BMI < 30, *n* = 14. The hypergeometric *P* values were adjusted for multiple testing using the Benjamini–Hochberg method. AUC, area under the curve. Source data

BMI and lifestyle factors have significant roles in testicular function, contributing to several aging trajectories and fertility outcomes in humans^9^. To elucidate the correlation between BMI and fertility and its interaction with age, we analyzed donor records. Interestingly, we noted that donors with higher BMI levels exhibited reduced spermatid counts, despite still being able to father offspring (Fig. 6a), indicating that while a certain BMI threshold may not cause infertility, it could confer subfertility. Subsequently, coefficient variance analysis encompassing age and BMI revealed that transcriptomic alterations associated with age were notably more prominent than those associated with BMI (Fig. 6b).

To further explore the influence of BMI and age on male fertility, predictive models for age or BMI were independently constructed based on the number of sperm using PAS staining in each donor. The results suggested that both aging (especially beyond 45 years) and elevated BMI (particularly exceeding BMI ≥ 30) were potential risk factors for subfertility (Fig. 6c). Furthermore, the frequency of haploid germ cells showed significant differences among different BMI groups, especially between BMI ≥ 30 and BMI < 30, indicating a pronounced interaction between BMI and aging, particularly at or after the age of 40, influencing fertility (Fig. 6d). Additionally, although BMI contributed to the influence of aging after 40 years, we also explored whether very high BMI itself is correlated with subfertility. To explore this, we stratified BMI into five subclasses, including normal (18–25), overweight (25–30), obesity I (30–35), obesity II (35–40) and obesity III (>40). The frequency of round and elongating spermatids exhibited a significant decline in class III obesity (BMI exceeding 40) (Fig. 6e).

Furthermore, to illustrate the relationship between BMI and age in somatic cells, we conducted differential gene expression analysis on high (BMI ≥ 30) and low (BMI < 30) BMI groups in their 30s and 50s compared to their 20s. The upregulated and downregulated pathways in TPCs, Leydig cells and SMCs revealed that the influence of high BMI is similar to age-related changes (Fig. 6f–h and Supplementary Fig. 9). Hence, BMI is strongly correlated with aging-related alterations. In summary, pronounced subinfertility was associated with a BMI exceeding 40 or an age surpassing 45, particularly with a BMI greater than 30.

## Discussion

The aging process in human adult testes is complex and many interesting questions remain unanswered, in particular the identification of the cell types that are first affected and the pathways involved. In this study, we conducted single-cell transcriptome profiling across the entire male reproductive spectrum. This approach serves as a model for integrating machine learning, which is widely used in several species^70,71^, with single-cell analysis to investigate key molecular genes and cell types. Additionally, the resulting dataset allows for the identification and visualization of key age groups in both somatic and germline cells. Furthermore, comprehensive single-cell analyses provide insights into identifying key ages and supporting evidence of both gradual changes and more acute onset in age-related alterations, as well as relationships to comorbidities, such as elevated BMI, in testis health. Together, these findings contribute to understanding how to maintain male reproductive health and identify optimal reproductive ages (Supplementary Fig. 10).

To elucidate the influence of aging on somatic cells, we focused on consistent and specific age-related changes. First, we observed a continuous increase in immune response across the ages, a common phenomenon during aging^23,72–74^, which was also evident in somatic cells. Second, we identified key age-related changes that occurred abruptly during aging. For instance, the pronounced increase in basement membrane thickness observed in TPCs displayed an onset in donors during their 30s, and reflected changes in DEGs enriched in ECM and collagen I pathways, requiring further validation of the relationship between collagen I and ECM signaling. These findings, supported by experimental validations, suggest TPCs as a priming cell type in responding to aging. Consistent with this notion, TPCs in donors in their 30s exhibited the highest variability coefficient, significant changes in gene expression and dynamic pathways across the ages. Specifically, genes related to the ECM (for example, *MMP14*, *COL14A1*, *COL5A2*, *COL5A1*, *COLGALT1*, *EFEMP2* and *APP*), and oxidative stress-related genes (for example, *HSPA1A*, *HSPA1B* and *CTNNB1*) all showed a decline in their expression in donors in their 30s, indicating a strong correlation between ECM and age. Targeting the ECM may have the potential to improve the aging process. Other significant events emerged in donors during their 50s involving Sertoli and Leydig cells. Sertoli cells displayed changes in nutrient-related genes^75^ (for example, *IGF1R*, *INSR*) and cell–cell communication, while Leydig cells exhibited changes in their ability to synthesize and metabolize testosterone. As the primary hormone-secreting cells in the testes, their fluctuations regulate the testicular microenvironment and spermatogenesis. Overall, our work revealed two waves of molecular and cellular changes during human testis aging. At the first wave, TPCs respond to aging with a lower threshold to ‘switch’ to ECM-related pathways, in contrast to other somatic cells, which exhibit minimal transcriptomic alterations. At the second wave, the cumulative response in somatic cells reaches a threshold where age-associated transcriptomic characteristics are significantly amplified so that inflammation^76,77^ and cluster-specific aging-related pathways, including testosterone production, muscle contraction and ECM, are identified as hallmarks for aging. Although an inability to capture cells because of apoptosis or other factors is a common limitation in all single-cell analyses, these results underscore how somatic cells exert specific influences on subfertility during aging.

Furthermore, we observed changes in germ cells at the molecular and cellular levels during aging. Although SSCs showed minimal transcriptome changes across the ages^9^, we noted a decrease in SSC counts in donors in their 50s, which is consistent with previous studies indicating that the transcriptome of SSCs from subfertile or infertile men, including patients with Klinefelter syndrome, resembles that of fertile men^78,79^. Notably, a significant increase in UTF1^+^ and cKIT^+^ cells was observed in donors in their 30s, as well as a significant increase in cKIT^+^ cells in donors aged from their 50s to their 60s. To explain such an observation, we highlight the distinct roles of SSCs and differentiating spermatogonia in supporting spermatogenesis. SSCs are the sole germline stem cells capable of self-renewal and differentiation into sperm, while differentiating spermatogonia are progenitor cells that rapidly proliferate and differentiate to enter meiosis. As a reservoir for male reproduction, SSCs respond gradually to the aging niche, whereas differentiating spermatogonia can quickly react to internal and external stimuli to maintain continuous spermatogenesis. Indeed, SSC numbers are highest during the 30s and 40s, coinciding with the peak reproductive years for adult men. The trend in differentiating spermatogonia closely follows that of SSCs. Notably, there is a significant increase in the number of differentiating spermatogonia in donors in their 60s, suggesting a potential need for greater spermatogonial amplification in older testes to sustain sperm production. However, spermatids exhibited dynamic changes in gene expression during aging, which suggest a cumulative response of germ cells to aging, which is most evident in donors during their 50s, correlating with an increase in sperm morphology and flagellar motility. Previous studies elucidated the functions of specific genes such as *QRICH2* (ref. ^62^), *FSIP2* (refs. ^66,67,80^), *CARM1* (ref. ^60^), *CENPJ*^65^ and *CEP152* (ref. ^68^), which may have crucial roles in mediating these age-related changes in spermatids through stabilizing and boosting the expression of proteins associated with flagellar development. Interestingly, our study did not detect a significant decrease in spermatid counts in the testes of older men. One possible explanation is that the typical process of differentiation into spermatids disrupted sperm metamorphosis. In the context of aging, the ability of flagellation and metamorphosis in round and elongating spermatids declines, resulting in the accumulation of abnormal spermatids in the testes, which is consistent with subfertility in older individuals exhibiting abnormal semen parameters^81^ and low sperm genome integrity^5,18,19^. Our findings suggest a cumulative effect of aging on sperm health and provide a deeper understanding of characteristics during aging. Furthermore, the cumulative effect of germ cells, which is consistent with sperm DNA damage, increased with age^82^. Alterations in gene expression reflect underlying changes in cellular function in germline cells. These changes may influence critical processes, such as DNA repair and epigenetic modifications^83,84^, all of which are essential for maintaining sperm genomic integrity and reproductive capacity. Understanding how gene expression profiles evolve with age can shed light on age-related reproductive decline, address aging-related fertility decline or diseases affecting male infertility, and offer potential therapeutic targets to improve male reproductive health.

Environmental and physiological traits influence spermatogenesis and subsequently offspring^85^. To further explore environmental and physiological traits during aging, we focused on BMI. While previous research delved into the complex mechanisms through which obesity affects male fertility, the connection between obesity and aging has not been extensively studied^86^. Cell frequency analysis supported the notion that higher BMI is highly associated with subfertility, while intermediate BMI has a significant cooperative role with age-related changes. Additionally, we found that BMI-related changes resembled age-related changes in somatic cells, suggesting that elevated BMI accelerates aging. Nevertheless, the precise mechanism through which BMI influences aging is a subject requiring further exploration, especially considering potential disruptions in the metabolic profiles of brain-dead donors. Despite the consistent aging-related transformations in somatic cells, a deeper understanding of the interplay between BMI and age necessitates additional research to elucidate the underlying mechanisms, ultimately contributing to advancements in male reproductive health.

To summarize, we created a comprehensive atlas of the human testis from 35 male donors, spanning the male reproductive lifespan, which serves as a valuable resource for investigating the impact of aging on spermatogenesis and the human testes. Our analysis identified two distinct waves in somatic cell types during aging and supported the cumulative effect of aging on haploid germ cells. Furthermore, by combining age and BMI data, we illustrated their relationship and highlighted the influence of environmental factors on aging.

## Methods

### Sample collection

All human testicular tissues were obtained through DonorConnect (formerly Intermountain Donor Services), a nonprofit community service organization in Salt Lake City dedicated to the recovery and transplantation of organs and tissues for Utah, southeastern Idaho, western Wyoming, and Elko, Nevada. These samples were removed from deceased individuals who had previously consented to organ donation for transplantation and research. For samples from deceased individuals, institutional review board approval is not applicable according to the National Institutes of Health Investigator Manual for Human Subjects Research.

### Sample transportation and storage

After surgical extraction, pairs of intact testes were placed in containers with ice for 1–2 h during transportation. Then, the tunica albuginea was removed and the testicular tissues were dissected into 500-mg pieces. Ninety percent of the dissected tissues were transferred into cryovials (Corning) prefilled with 1.5 ml of freezing medium consisting of 75% DMEM (Thermo Fisher Scientific), 10% dimethyl sulfoxide (cat. no. D8779, Sigma-Aldrich) and 15% FCS (Gibco). These cryovials were then placed in an isopropanol chamber (Thermo Fisher Scientific) and subjected to gradual freezing at −80 °C overnight. Subsequently, the cryovials were transferred to liquid nitrogen for long-term preservation.

### Sample fixation

Around 10% of testicular tissues were fixed in 1× Dulbecco’s PBS (DPBS) (Thermo Fisher Scientific) containing 4% paraformaldehyde (cat. no. 28908, Thermo Fisher Scientific) and agitated overnight at 4 °C and 60 rpm. The fixed samples were thoroughly washed three times with cold PBS and subsequently stored in PBS at 4 °C until further processing for immunostaining or histochemistry.

### PAS staining

Fixed tissues were embedded in paraffin and cut by 0.4 mm per section. Sections were deparaffinized and hydrated to water. Then sections were oxidized in 0.5% periodic acid solution for 5 min, incubated in Schiff’s reagent for 15 min and counterstained in Mayer’s hematoxylin for 1 min. Between each step, the sections were rinsed in distilled water. Finally, the sections were dehydrated and mounted using xylene-based mounting medium.

### IF staining

Fixed samples were then washed three times in PBS before being embedded in paraffin. Formalin-fixed paraffin-embedded samples were cut into 4-μm sections. Sections were deparaffinized using CitriSolv (cat. no. 1601, Decon Labs) and rehydrated in an ethanol series (100%, 95%, 80%, 70%, water). Then, sections were microwaved for 20 min in 1 mM EDTA (pH, 8.0) or EDTA antigen retrieval buffer (pH, 8.0) (cat. no. ab93680, Abcam) for antigen retrieval. After cooling down, sections were incubated with blocking buffer (5% donkey serum, 3% BSA in PBS) at room temperature for 1 h. Then, individual sections were incubated with diluted primary antibodies (UTF1, 1:1,000 dilution in PBS; cKIT, 1:1,000 dilution in PBS; collagen I, 1:1,000 dilution in PBS) at 4 °C overnight in a humidified chamber. Sections were washed with PBS and incubated with secondary antibodies at room temperature for 1 h followed by PBS washes. DAPI was added (1:300 dilution in PBS) to the sections for 2 min at room temperature for counterstaining followed by PBS washes. Finally, sections were mounted using the ProLong Gold Antifade Mountant (cat. no. P36930, Invitrogen) and sealed using clear nail polish. Images were acquired with a Leica SP8 confocal microscope.

### scRNA-seq

For each scRNA-seq experiment, one cryovial containing tissue was rapidly thawed and washed with PBS; the contents were then enzymatically digested to yield a single-cell suspension. Briefly, tissues were scraped with razor blades and digested with a mixture of 1 mg ml^−1^ collagenase type IV (Sigma-Aldrich) and 1 mg ml^−1^ DNase I (Sigma-Aldrich) at 37 °C for 5 min. The medium was then filtered through 70-μm strainers (Thermo Fisher Scientific) and the flow-through cells were washed with DPBS. The remaining tissue chunks were subjected to further treatment with trypsin-EDTA (Invitrogen) and 1 mg ml^−1^ DNase I for another 5 min. The resulting single cells were filtered through 40-μm strainers and washed with DPBS. Finally, cells from each digestion step were combined and resuspended in DPBS containing 0.4% BSA (Thermo Fisher Scientific) at a concentration of 1,000 cells ml^−1^ for scRNA-seq.

The procedures were guided by the user manual of the Chromium Next GEM Single Cell 3′ Reagent Kits v3.1, developed by 10x Genomics. Briefly, for the GEM Generation and Barcoding step, cells were diluted to recover 5,000 cells per lane and then loaded with master mix on the Chromium Next GEM Chip G. After Post GEM-RT Cleanup, 12 cycles were used for complementary DNA amplification. The resulting libraries were then sequenced on an Illumina NovaSeq instrument with the following settings: 28 cycles for read 1; ten cycles for the i5 index; ten cycles for the i7 index; and 90 cycles for read 2.

### Data processing and quality control

CellRanger^87^ v.2.2.0 was used to demultiplex raw data using the mkfastq function. The output FASTQ files were then run with function count using default settings, including alignment to the GRCh38 human reference genome. Subsequently, the resulting matrices were imported into R v.4.2.2, with exclusion criteria applied to remove features expressing fewer than 200 genes and genes expressed in fewer than three cells. When publicly available data were reanalyzed, raw cell/count matrices were downloaded and reprocessed using the aforementioned workflow.

In our downstream analysis, we merged our sequencing data with published data to perform quality control. Features with fewer than 200 or more than 5,500 features, UMIs with fewer than 800 or more than 17,000 counts and cells with greater than 25% mitochondrial mapping were filtered out. Subsequently, integration of the datasets exhibited little batch effect at different ages. Principal component and UMAP analyses were performed based on 5,000 high variable genes. Each cluster was annotated with the top 20 genes obtained using the FindAllMarkers function with default parameters.

### Cluster ratio and frequency analyses

The cluster ratio analysis was conducted using the ggplot2 package. A bar plot illustrating the changes in cluster ratios across different age groups was generated using ggplot2. The cluster frequency analysis was derived from the cluster ratio, with the frequency calculated as the ratio multiplied by 100.

### DEG and functional enrichment analyses

We divided ages into 20s (20–29), 30s (30–39), 40s (40–49), 50s (50–59) and 60s (60–69) and performed pairwise comparisons according to age group. Differential gene expression analysis was performed using the FindMarkers function in Seurat^88,89^ based on Wilcoxon rank-sum test. Genes were considered significant if *P* < 0.05 and the log_2_ fold change was greater than 0.25 or less than −0.25.

Significant DEGs for each cluster and pairwise groups were inputted for the GO analysis using clusterProfiler^90^. We selected the biological process pathways and *P* < 0.05, and a gene count greater than 2, as significant pathways.

### Cell–cell communication analysis

Cell–cell communication was inferred through CellChat^91^. The CellChatDB.human database was specifically chosen for this analysis. The expression matrix served as the input for CellChat using default settings (type = triMean, min.cells = 10). After this, we extracted cell–cell communication counts and weights and the inferred cell–cell communication through the netVisual_circle and subsetCommunication functions. To visualize the differential number of interactions or interaction strength across distinct cell populations, comparison of interaction counts and weights between age groups were conducted and visualized using the igraph R package.

### Mfuzz analysis

To identify dynamic patterns along ages, we conducted the Mfuzz analysis^92^. Genes exhibiting an average expression below 0.1 in each age group were excluded from the analysis. Subsequently, expression matrices were subjected to clustering using the fuzzy C-means algorithm.

### GSVA analysis

To identify how genes respond to age-related changes, we performed GSVA^93^. Genes with an average expression greater than 0.1 were used as input for the GSVA analysis. We estimated GSVA enrichment scores using a Gaussian kernel. Furthermore, we conducted a limma^94^ analysis to find significant (*P* < 0.05 and log_2_ fold change > 0.5) pathways using a Bayes test. Subsequently, we selected representative pathways and fitted GSVA scores along ages to visualize the data.

### Age prediction using machine learning

We divided the age groups into five classifications: 20s, 30s, 40s, 50s and 60s, and selected testicular cell types for age prediction. We used five categorical features; filtered features detected in more than 200 cells and with a variance inflation coefficient greater than ten were removed to eliminate collinearity between features for machine learning. We used Scikit-learn^95^ to create eight models to predict ages.

For each model, 80% of cells were split into training and validation sets using fivefold cross-validation; the remaining 20% of cells were split into the test set. StandardScaler was applied for preprocessing. GridSearchCV was used to search for the best parameters according to the accuracy of the model in the test set. Each model was retrained over ten random states with the best parameters. For prediction accuracy, the XGBoost classifier^56^ was chosen and used with the Matthews correlation coefficient and Kappa coefficient, as the level of response to senescence for each cell type. Model interpretation was performed using the importance of permutated features.

### TE analysis

To illustrate the dynamic expression of TEs during aging, we used scTE^59^ to obtain the TEs. First, the human repeat masker (RMSK) file was downloaded from the UCSC database to annotate the TEs. The BAM files, generated by CellRanger, were filtered based on the selected barcodes. The cleaned BAM files, characterized by the inclusion of barcodes in the ‘CB:Z’ column, were processed using scTE and default parameters (--hdf5 True -CB CR -UMI UR). The resultant files—single-cell expression data in H5ad format—were then transformed into Seurat objects to perform different expression gene analysis.

### SCENIC analysis

To identify key regulons and further explore the regulatory network, we conducted a pySCENIC analysis^69,96^. The human cisTarget database was downloaded from https://resources.aertslab.org/cistarget. A three-step approach was used. First, we converted the expression matrix to the loom and used the pyscenic grn, pyscenic ctx and pyscenic aucell functions to infer the gene regulatory network, predict regulon activity and identify the gene regulatory networks associated with major transcription factors and their target genes for each cell type. RSS were calculated in each cluster. The gene regulatory network was visualized using the igraph R package.

### Scater analysis

To identify the key factors influencing the variability in expression data among cells, we used scater^97^ to assess the explained variance associated with variables such as age and BMI. The expression matrix, encapsulated in the SCESet object, was normalized using the logNormCounts function. Subsequently, individual linear models were constructed for all genes, with the explanatory variables through the getVarianceExplained function. After this, the distribution of explanatory variables was visually represented using the plotExplanatoryVariables function. Statistical analysis was performed using the Wilcoxon (rank-sum) test.

### ROC analysis

To predict infertility and determine the optimal cutoff value of ages and BMI, we created ROC curves using multipleROC^98^. The prediction model was developed based on the sperm numbers detected through PAS staining, with infertility defined as a sperm count of fewer than three per seminiferous tubule. The ROC plot with sensitivity, specificity, positive predictive value, negative predictive value and optimal cutoff value was visualized using the multipleROC function.

### Cell counting and basement membrane measurement

To facilitate the quantification of basement membrane thickness, a total of 200 seminiferous tubules in each sample with PAS staining were selected to perform the statistical analysis. Basement membrane thickness was assessed by measuring the width of the basement membrane in a direction perpendicular to the orientation of the seminiferous tubules, using ImageJ for the measurements.

To quantify the expression of UTF1 and cKIT, cells exhibiting coexpression of DAPI and UTF1/cKIT were evaluated on the transverse sections of circular seminiferous tubules. For each sample, 25 tubules were evaluated, with each experimental group including two distinct samples subjected to staining.

To quantify the expression of collagen I, a strategic selection was made to include regions both external and internal to the seminiferous tubules. Subsequently, regions extracted from the differential set were used to compute the mean IF intensity using ImageJ, thereby serving as a representative measure of collagen I expression. For each sample, 15 tubules were selected, with each age group including two distinct samples subjected to staining.

Box plots were used to depict the distribution of parameters across individual tubules. Statistical analysis of the data was performed using an unpaired, two-sided Student’s *t*-test or a Wilcoxon rank-sum test, facilitated by the ggsignif package. *P* < 0.05 was deemed statistically significant.

### Statistics and reproducibility

No statistical method was used to predetermine sample size, but our sample sizes were comparable to those reported in previous studies^22,72^. No randomization was used. Data collection and analysis were not conducted in a blinded manner with respect to the experimental conditions. Low-quality cells were excluded from the scRNA-seq analysis based on pre-established quality control criteria to ensure the reliability of the data. Data distribution was assumed to be normal but this was not formally tested. Statistical analyses were performed in R (v.4.2.2), with the appropriate statistical tests applied. Further details on the statistical methods can be found in the relevant subsections of the Results and in the figure legends.

### Reporting summary

Further information on research design is available in the Nature Portfolio Reporting Summary linked to this article.

## Supplementary information

Supplementary Information Supplementary Figs. 1–10.

Reporting Summary

Supplementary Tables 1–3 Supplementary Table 1: Percentage of cell types in each age. Supplementary Table 2: Accuracy of ten random states in each cell type according to the XGBoost model. Supplementary Table 3: Top 30 permutated features in each cell type using the XGBoost model.

## Source data

Source Data Fig. 1 Statistical source data.

Source Data Fig. 2 Statistical source data.

Source Data Fig. 3 Statistical source data.

Source Data Fig. 4 Statistical source data.

Source Data Fig. 5 Statistical source data.

Source Data Fig. 6 Statistical source data.

Source Data Extended Data Fig. 1 Statistical source data.

Source Data Extended Data Fig. 2 Statistical source data.

Source Data Extended Data Fig. 3 Statistical source data.

Source Data Extended Data Fig. 4 Statistical source data.

Source Data Extended Data Fig. 5 Statistical source data.

Source Data Extended Data Fig. 6 Statistical source data.

## Data Availability

The raw and processed data have been deposited in the Gene Expression Omnibus (GEO) under accession no. GSE254315. All data were analyzed with standard programs and packages. The published datasets analyzed for this study were downloaded from the GEO repository (accession nos. GSE182786 (ref. ^9^) and GSE215754 (ref. ^22^)) and reprocessed. The CellchatDB.human database was obtained through https://github.com/sqjin/CellChat. Additional information required to reanalyze the data reported in this article is available from the lead contact upon reasonable request.

## Code availability

All code used can be downloaded from https://github.com/JingtaoLab/Testicular_Aging.
