# Supplementary material for: Single-cell transcriptomic atlas of the human testis across the reproductive lifespan
Source: Nat Aging. 2025 Mar 3;5(4):658–74. doi: 10.1038/s43587-025-00824-2 (PMC12003174; doi:10.1038/s43587-025-00824-2)
Supplement: Supplementary file 1 — Supplementary Figs. 1–10. [file 43587_2025_824_MOESM1_ESM.pdf]

nature aging

Supplementary information https://doi.org/10.1038/s43587-025-00824-2

Single-cell transcriptomic atlas of the human testis across the reproductive lifespan

In the format provided by the authors and unedited

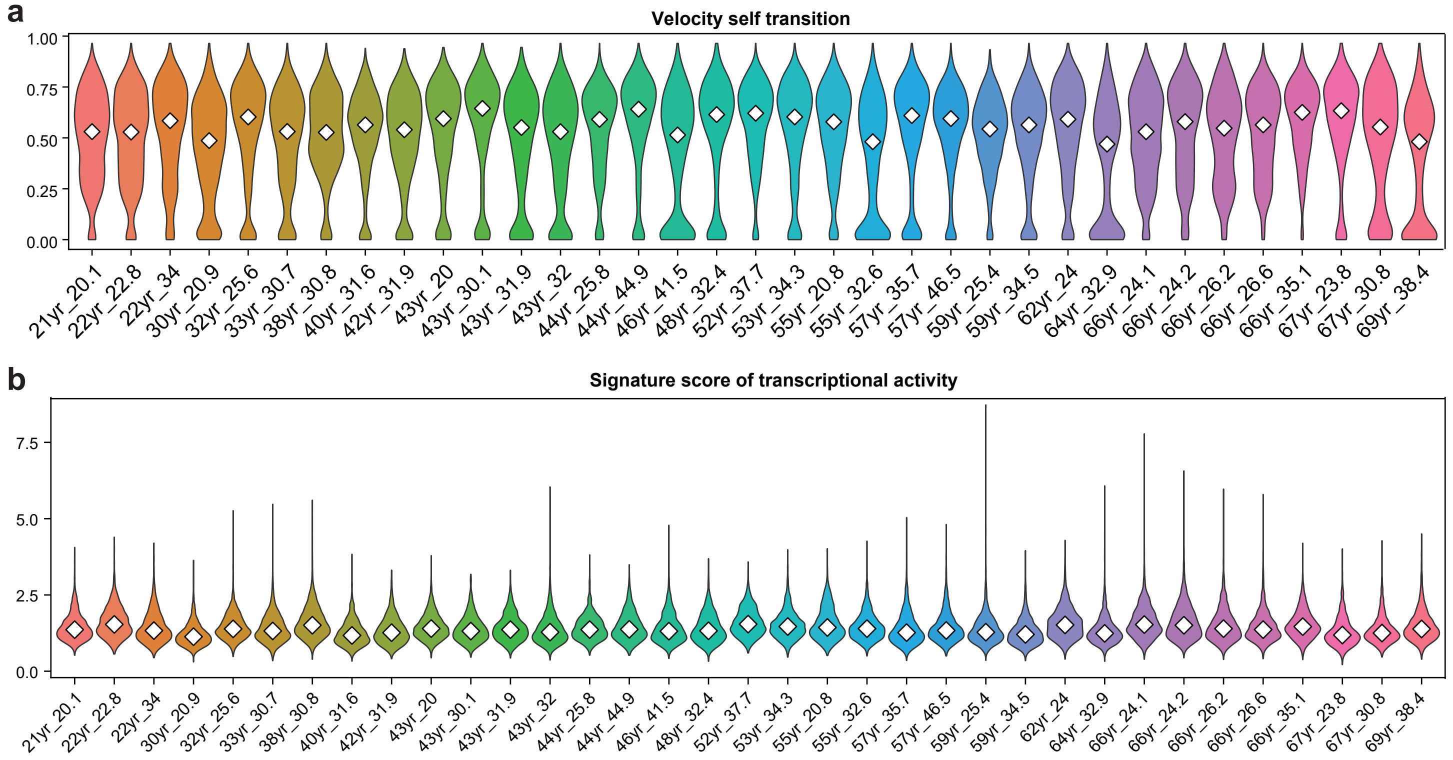

Supplementary Figure 1. The distribution of transcriptional activity in each donor.

a, Violin plot showing the distribution of velocity in each donor by scVelo analysis. **b**, Violin plot showing the distribution of signature score of transcriptional activity in each donor. Diamonds inside the violin represent the mean.

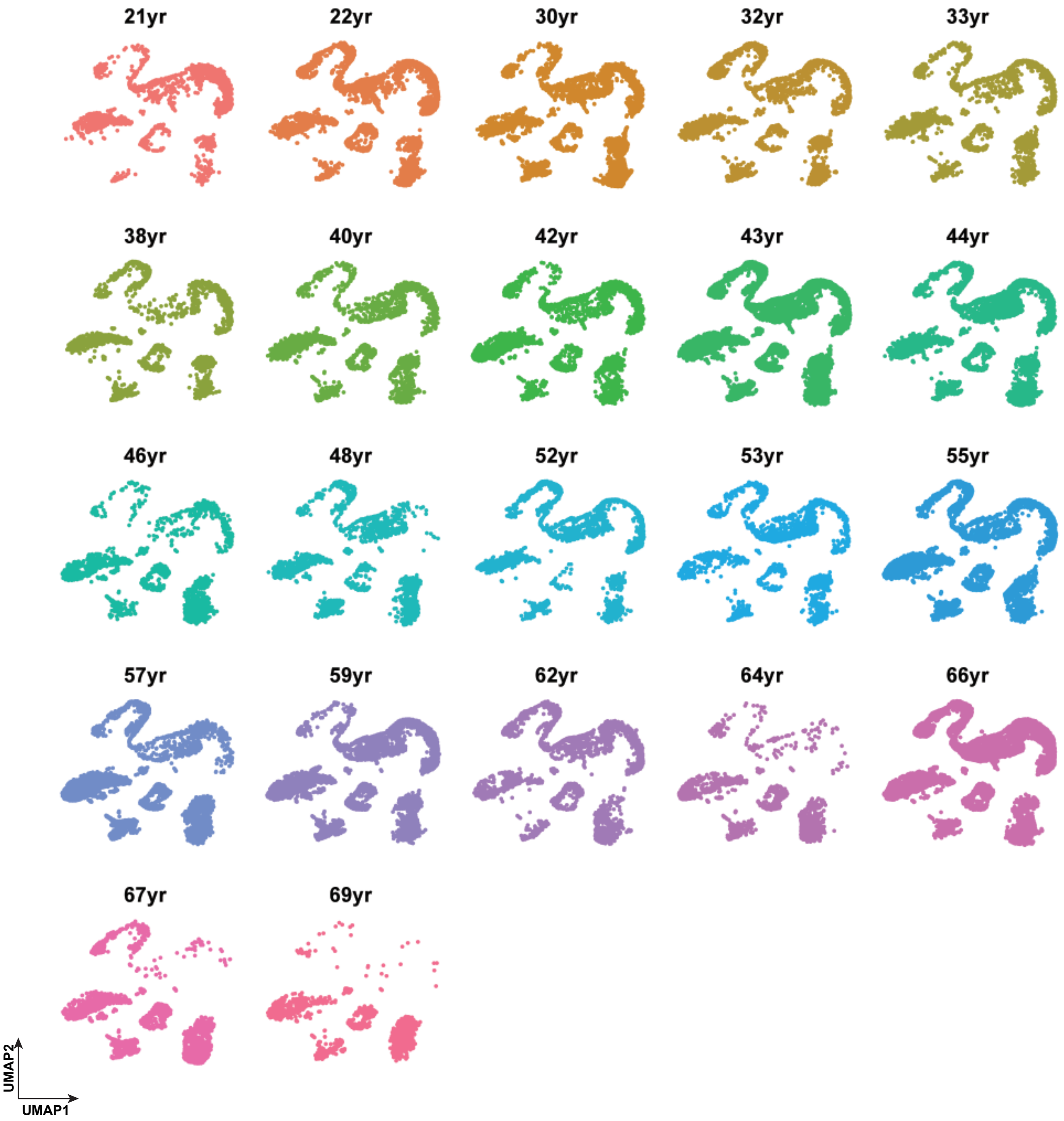

Supplementary Figure 2. Deconvolution of the UMAP plots according to ages in human testes.

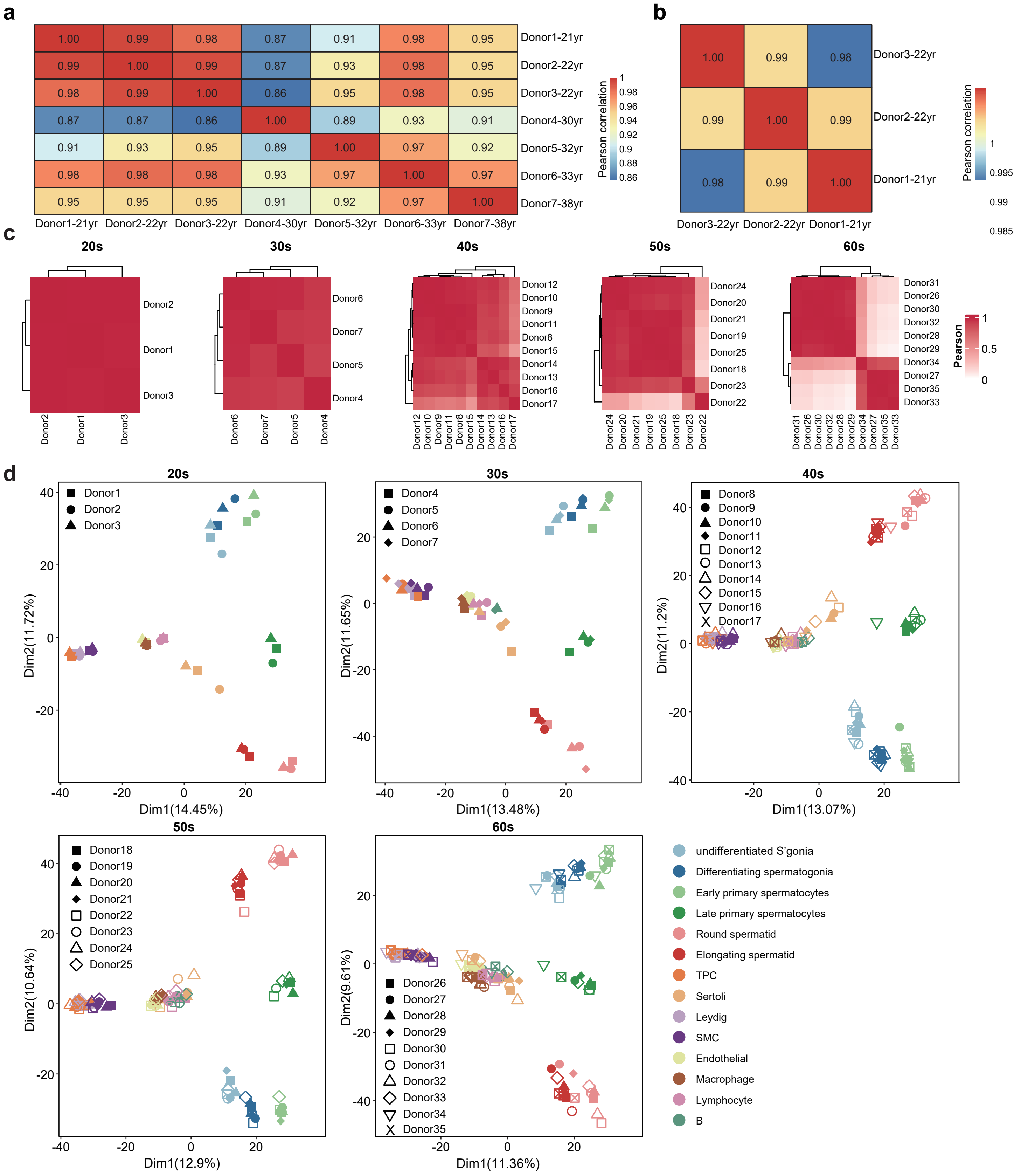

Supplementary Figure 3. The correlation of samples in each age group.
a, Heatmap plot showing the Pearson correlation of the samples in 20s. **b**, Heatmap plot showing the Pearson correlation of the samples in 20s and 30s. **c**, Heatmap of Pearson correlation between each age group. **d**, PCA plots showing the distributions of testicular cell types in each donor.

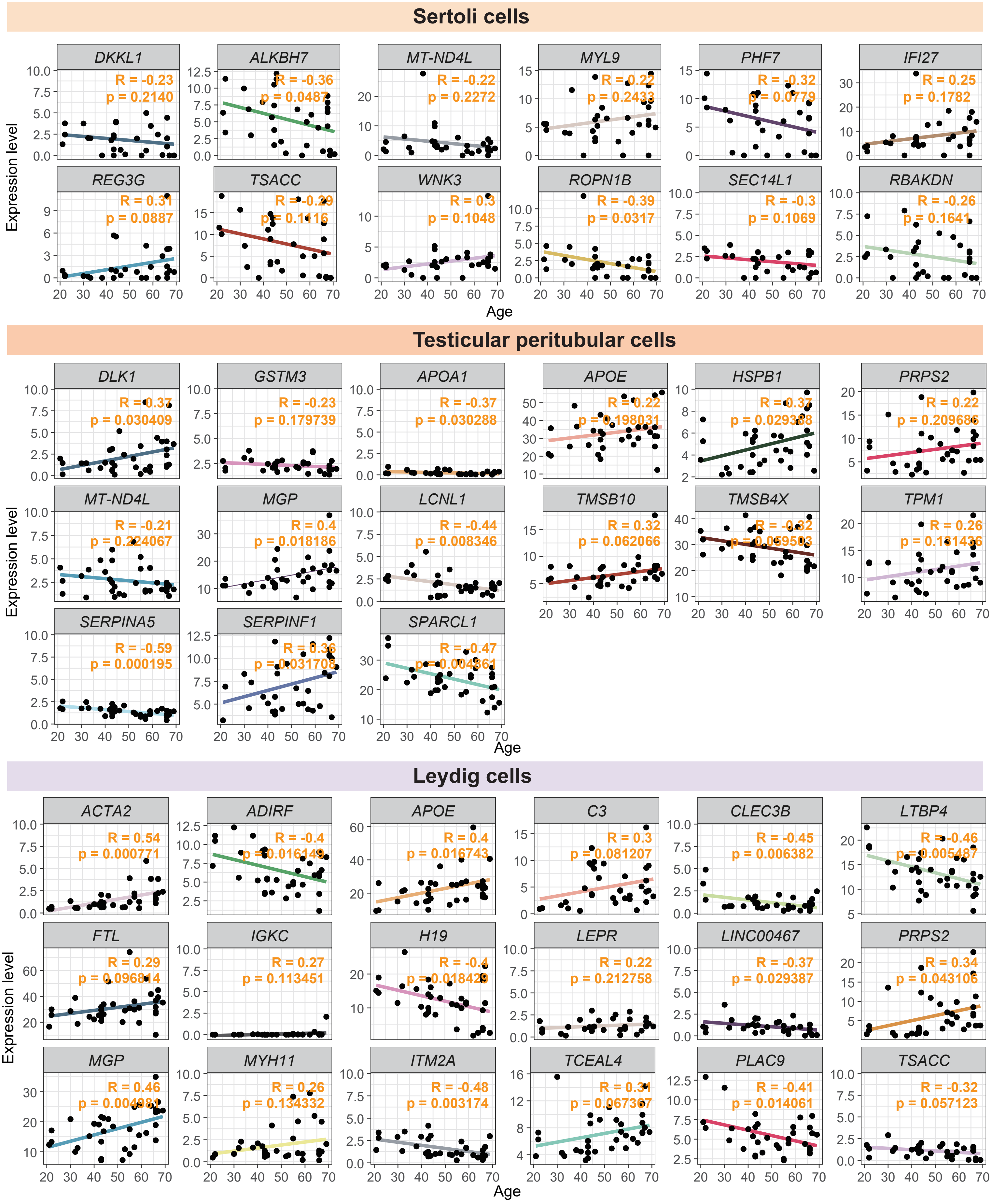

Supplementary Figure 4. Linear changing molecules during human aging.
The pearson correlation (R) highly correlate with the beta coefficients from linear regression models for Sertoli, testicular peritubular cells and Leydig cells. Black dots show the expression level and age of indivuduals (n=35). Permutation test; All P values presented are unadjusted.

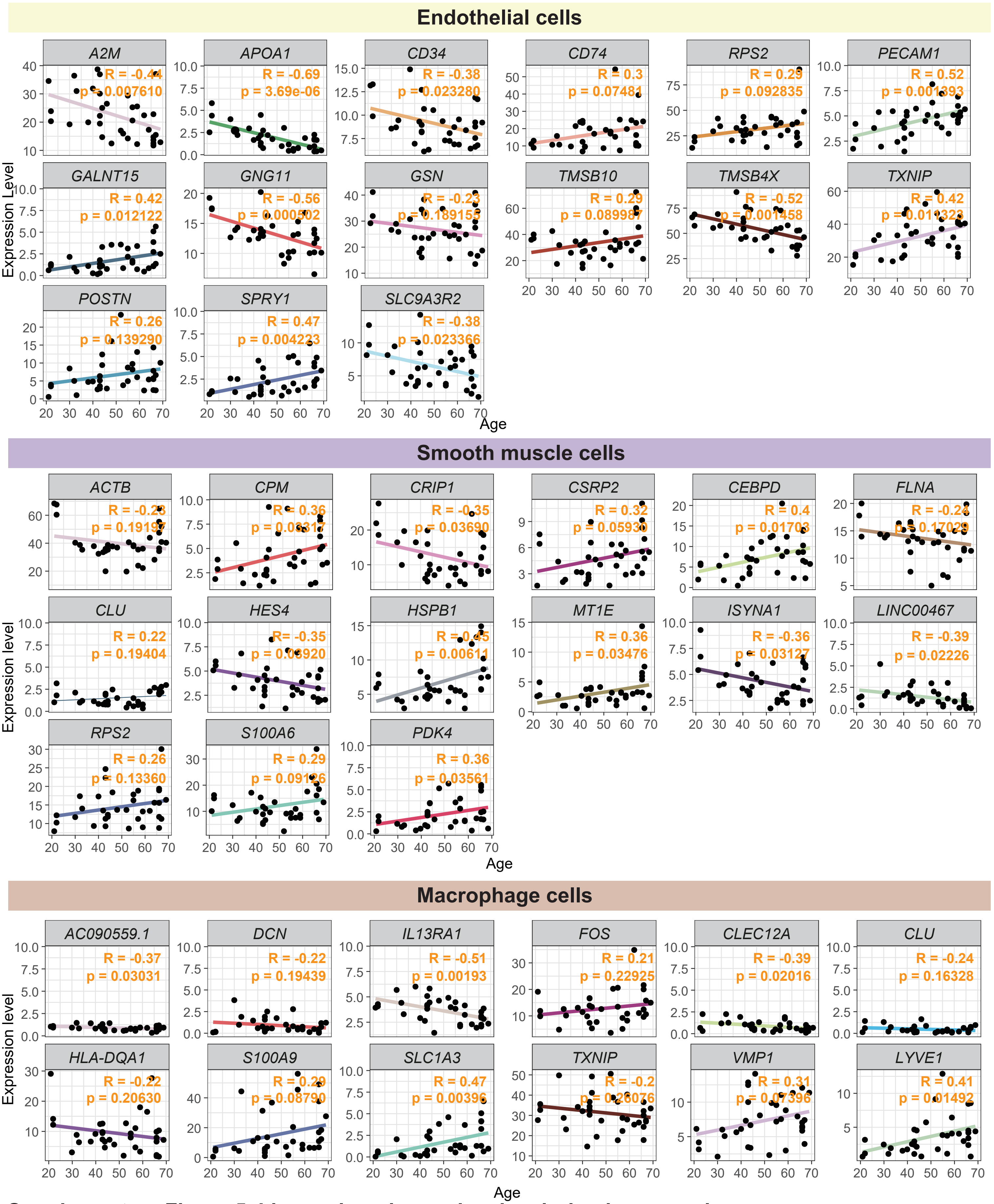

Supplementary Figure 5. Linear changing molecules during human aging.
The pearson correlation (R) highly correlate with the beta coefficients from linear regression models for endothelial cells, smooth muscle cells and macrophage. Black dots show the expression level and age of induviduals (n=35). Permutation test; All P values presented are unadjusted.

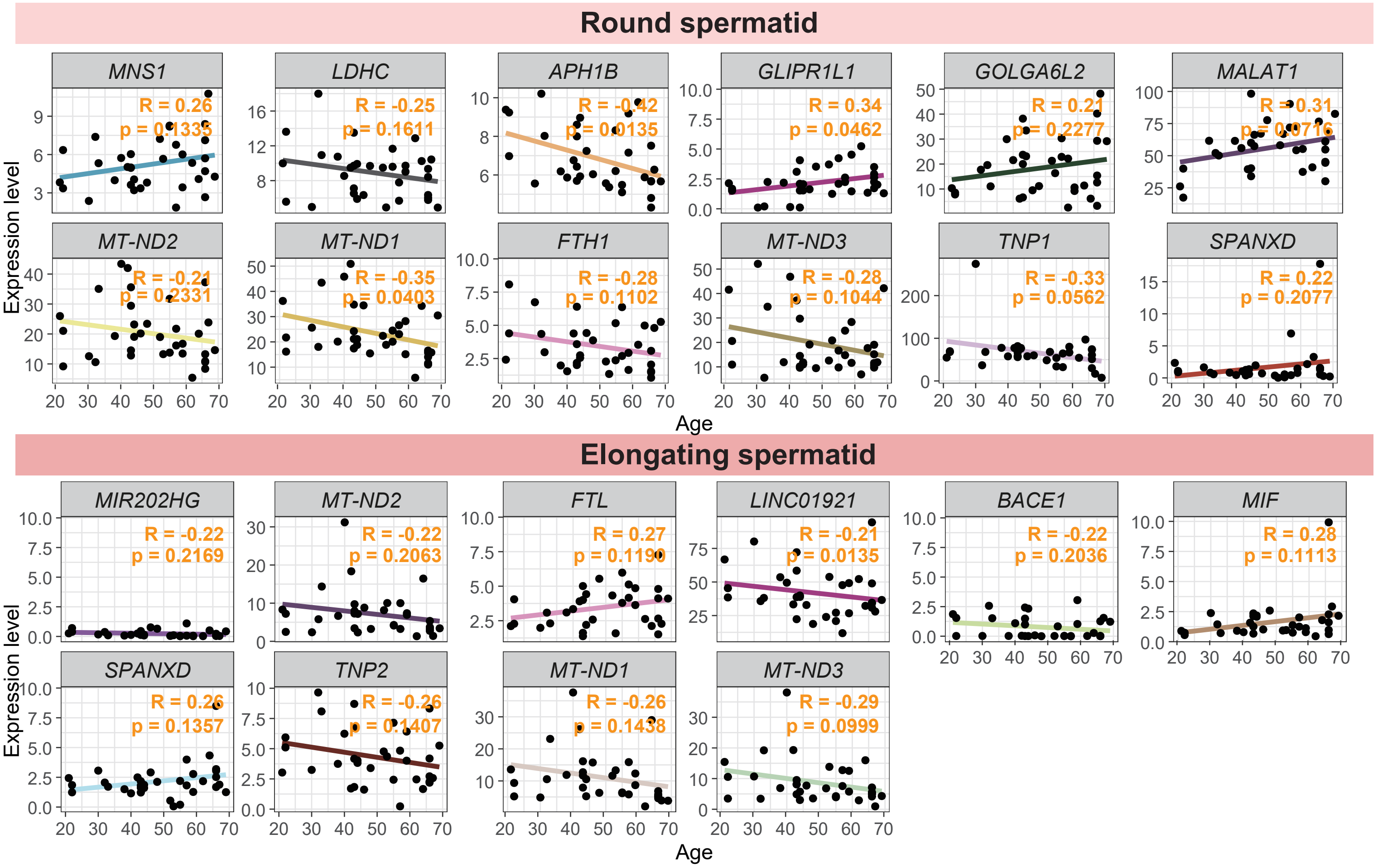

Supplementary Figure 6. Linear changing molecules during human aging.

The pearson correlation (R) highly correlate with the beta coefficients from linear regression models for round speramtid and elongating speramtid. Black dots show the expression level and age of indivuduals (n=35). Permutation test; All P values presented are unadjusted.

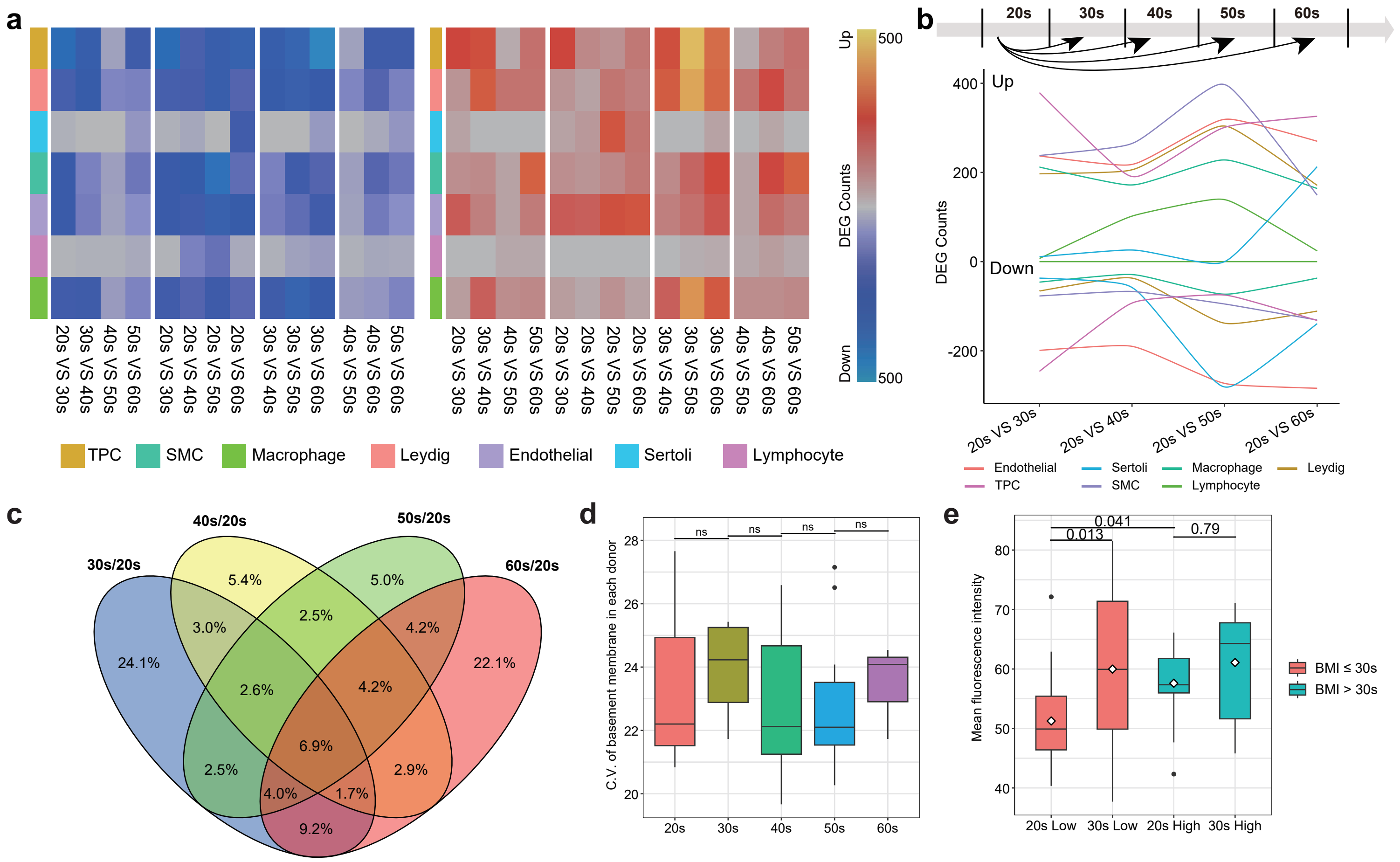

Supplementary Figure 7. The similarity and difference among the donors and clusters.
a, Heatmap plots showing the distribution of DGEs in pairwise comparison in somatic cells. **b**, Line plots showing the distributions of DEGs counts in somatic cells. **c**, Venn diagram showing the distribution of DEGs in TPCs based on randomly selected four samples from individuals in their 40s, 50s, and 60s. **d**, Boxplot showing the C.V. distribution of basement membrane. In almost all subtypes the c.v. is significantly changed in the 30s or 50s (two-sided wilcoxon test with Bonferroni correction). Box indicates range from 25th to 75th percentile, with whiskers extending to 1.5 times the interquartile range. Outliers are plotted separately; center indicates median value. Sample sizes: 20s, n=3; 30s, n=4; 40s, n=10; 50s, n=8; 60s, n=10. 20 testicular tubules from each donor are shown. Each age group include high BMI and low BMI individuals. **e**, Boxplot showing the mean fluorescence intensity of Collagen I in each tube (n=15). Diamond inside the violin represents the mean.Two-sided t-test with Bonferroni correction, P values are indicated. Whisker box plots show median, lower and upper quartiles and whiskers extending to 1.5 times the interquartile range.

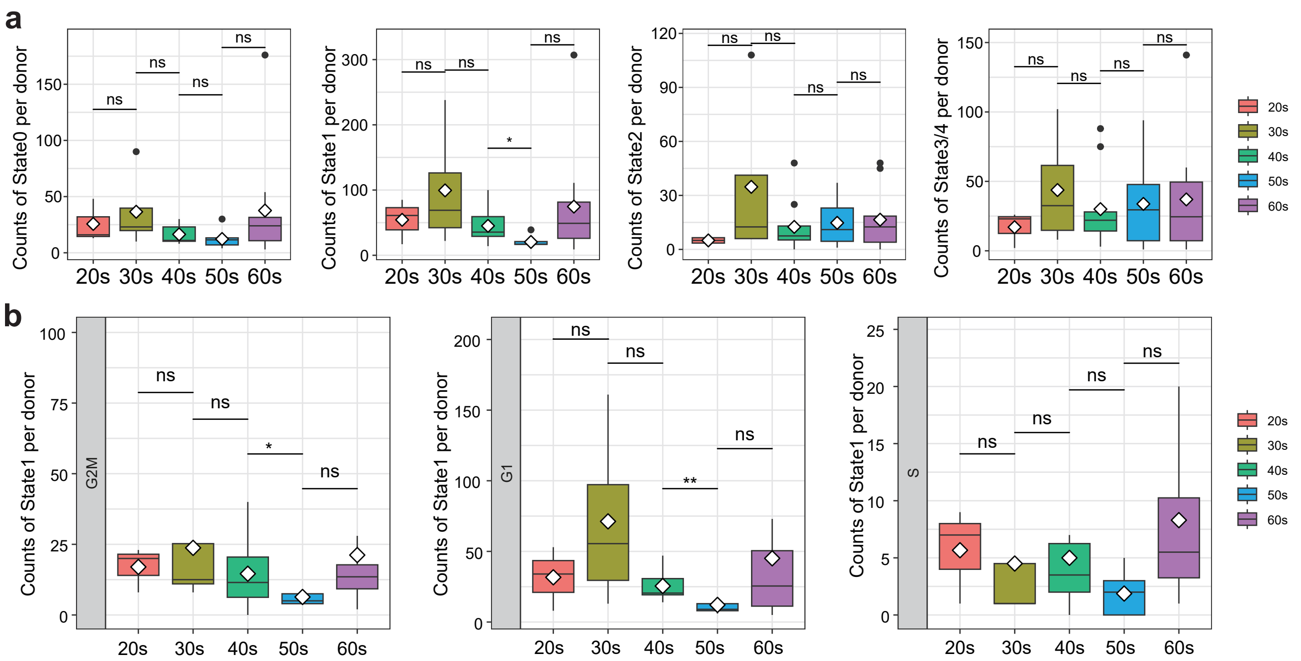

Supplementary Figure 8. The subtypes of spermatogonia.

a, Boxplots showing the dirtribution of cell counts detcted from scRNA-seq in each donor (n=35). Diamond inside the violin represents the mean. Two-sided t-test with Bonferroni correction, ns, p > 0.05. Whisker box plots show median, lower and upper quartiles and whiskers extending to 1.5 times the interquartile range. **b**, The boxplots shwoing the distribution of G2M, G1, S phases by cell cycle analysis in State1 subtype (n=35). Diamond inside the violin represents the mean. Two-sided t-test with Bonferroni correction, **, p <= 0.01; *, p <= 0.05; ns, p > 0.05. Whisker box plots show median, lower and upper quartiles and whiskers extending to 1.5 times the interquartile range.

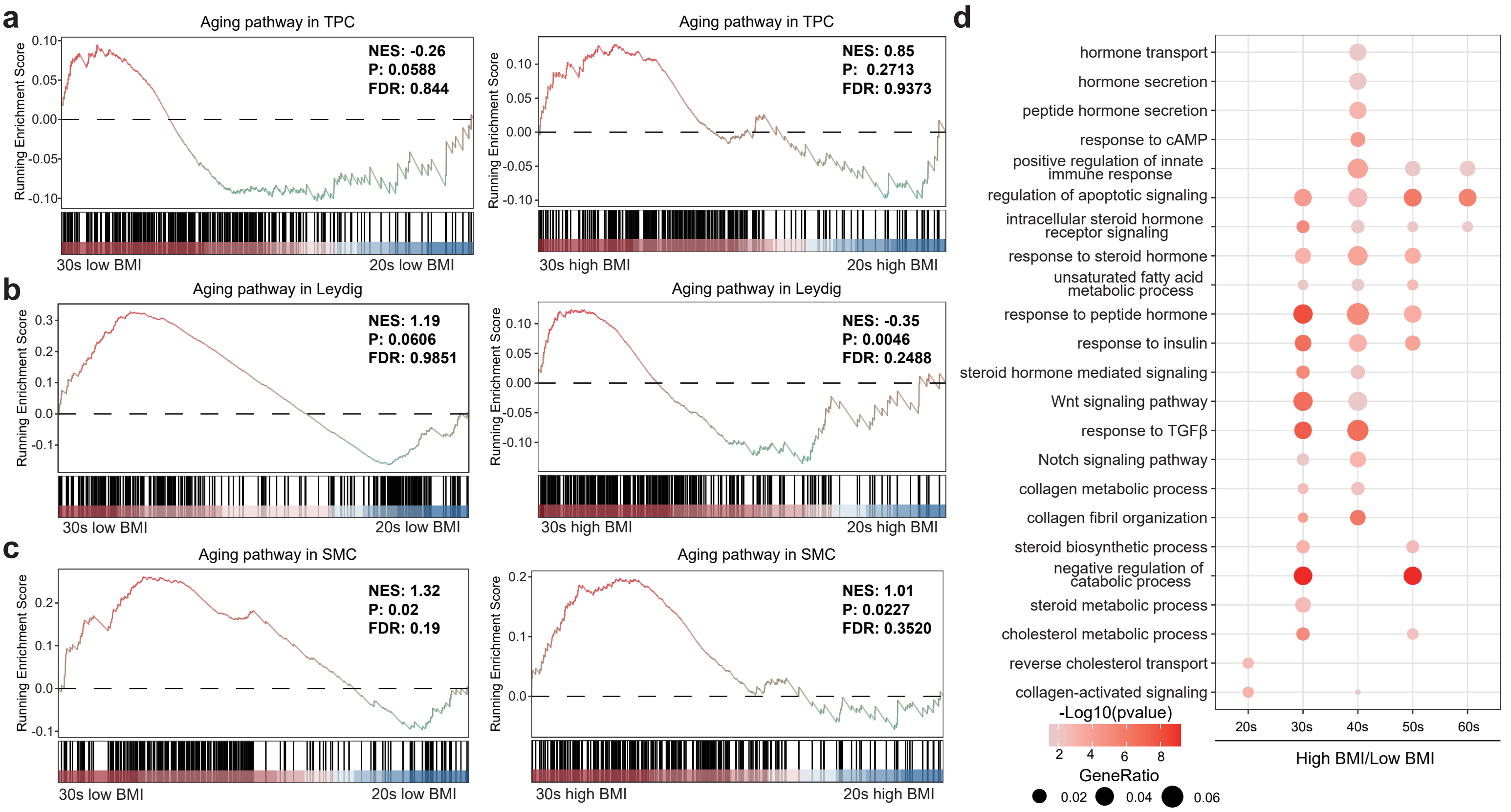

Supplementary Figure 9. The enrichments of somatic cells in high/low BMI.

a-c, the enrichment plots for the aging pathway in the TPC (a), Leydig (b), and SMC (c) by GSEA analysis. Permutation test with multiple comparisons adjusted by the Benjamini–Hochberg method. P values are indicated. **d**, Bubble plot illustrating the enrichments of DEGs from individuals with low BMI and high BMI in each age group in Leydig cells. The hypergeometric P values were adjusted for multiple testing using the Benjamini–Hochberg method.

Two waves of age-related/BMI-related changes in testis

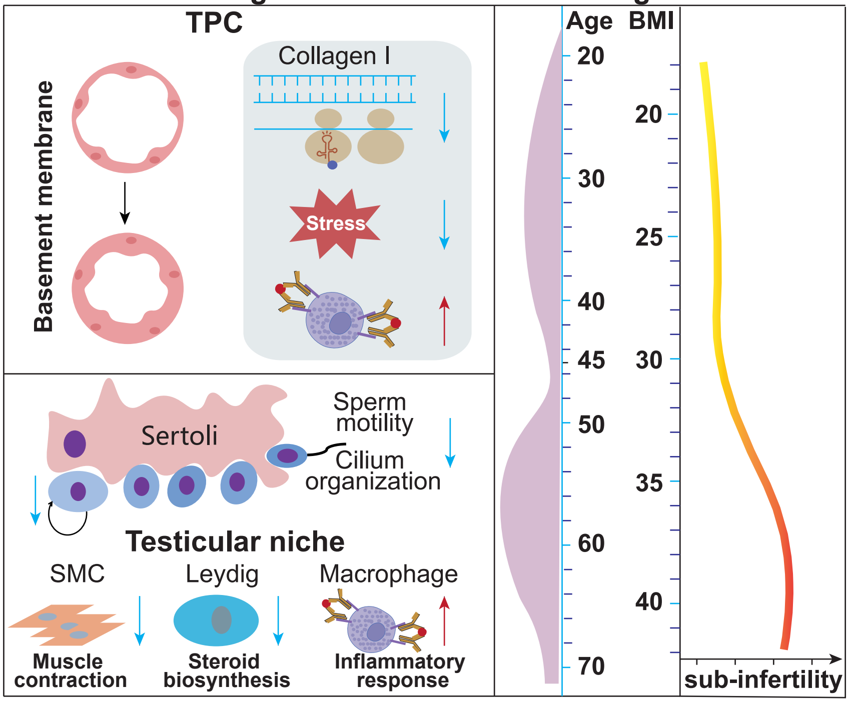

Supplementary Figure 10. Summary schematic summarizing the testicular changes at the molecular and cellular levels for donors in their 30s and 50s as well as the impact of BMI. The thickness of the basement membrane of the seminiferous tubules composed of testicular peritubular cell (TPCs) rapidly increases in the 30s, along with alterations in collagen I synthesis, oxidative stress, and immune response within the TPCs. By the age of 50s, there is a significant decrease in the number of spermatogonia cells, as well as a decline in sperm motility and cilium organization, and dysregulation of the functions responsible for normal spermatogenesis within the somatic cells. On the right side, the focus is mainly on the number of haploid germ cells and predictive models to demonstrate age- and BMI-related changes in spermatogenic capacity and specific variations. The arrows in blue/red indicate down/up.
