# Supplementary material for: Single-cell transcriptomic atlas of the human testis across the reproductive lifespan
Source: Nat Aging. 2025 Mar 3;5(4):658–74. doi: 10.1038/s43587-025-00824-2 (PMC12003174; doi:10.1038/s43587-025-00824-2)
Supplement: Supplementary file 2 — Reporting Summary [file 43587_2025_824_MOESM2_ESM.pdf]

nature portfolio

Corresponding author(s): Jingtao Guo, Bradley R. Cairns

Last updated by author(s): Jan 25, 2025

Reporting Summary

Nature Portfolio wishes to improve the reproducibility of the work that we publish. This form provides structure for consistency and transparency in reporting. For further information on Nature Portfolio policies, see our Editorial Policies and the Editorial Policy Checklist.

Statistics

For all statistical analyses, confirm that the following items are present in the figure legend, table legend, main text, or Methods section.

n/a	Confirmed	
☐	☒	The exact sample size (*n*) for each experimental group/condition, given as a discrete number and unit of measurement
☐	☒	A statement on whether measurements were taken from distinct samples or whether the same sample was measured repeatedly
☐	☒	The statistical test(s) used AND whether they are one- or two-sided<br>*Only common tests should be described solely by name; describe more complex techniques in the Methods section.*
☒	☐	A description of all covariates tested
☐	☒	A description of any assumptions or corrections, such as tests of normality and adjustment for multiple comparisons
☐	☒	A full description of the statistical parameters including central tendency (e.g. means) or other basic estimates (e.g. regression coefficient) AND variation (e.g. standard deviation) or associated estimates of uncertainty (e.g. confidence intervals)
☐	☒	For null hypothesis testing, the test statistic (e.g. *F*, *t*, *r*) with confidence intervals, effect sizes, degrees of freedom and *P* value noted<br>*Give P values as exact values whenever suitable.*
☒	☐	For Bayesian analysis, information on the choice of priors and Markov chain Monte Carlo settings
☒	☐	For hierarchical and complex designs, identification of the appropriate level for tests and full reporting of outcomes
☐	☒	Estimates of effect sizes (e.g. Cohen's *d*, Pearson's *r*), indicating how they were calculated

Our web collection on statistics for biologists contains articles on many of the points above.

Software and code

Policy information about availability of computer code

Data collection	Single-cell RNA seq data were sequenced on Illumina Novaseq.
Data analysis	All scRNA-seq data were processed with pySCENIC v0.12.1, R v4.2.2, Rstudio v2023.06.1-524 and the following R package were used: devtools v2.4.5, pheatmap v1.0.12, patchwork v1.1.2, ggplot2 v3.4.1, Seurat v4.3.0, SeuratDisk v0.0.0.9020, ComplexHeatmap v2.14.0, CellChat v2.0.0, clusterProfiler v4.6.2, Org.Hs.eg.db v3.16.0, factoextra v1.0.7, multipleROC v0.1.0, scater v1.30.1, Mfuzz v2.66.0, CellRanger v2.2.0.

For manuscripts utilizing custom algorithms or software that are central to the research but not yet described in published literature, software must be made available to editors and reviewers. We strongly encourage code deposition in a community repository (e.g. GitHub). See the Nature Portfolio guidelines for submitting code & software for further information.

Data

Policy information about availability of data

All manuscripts must include a data availability statement. This statement should provide the following information, where applicable:

- Accession codes, unique identifiers, or web links for publicly available datasets
- A description of any restrictions on data availability
- For clinical datasets or third party data, please ensure that the statement adheres to our policy

The raw data and processed data have been deposited in the GEO and the accession number is GEO: GSE254315. All data were analyzed with standard programs

and packages. The published datasets analysed for this study were downloaded from GEO repository (GSE182786 and GSE215754) and re-processed. All data were analyzed with standard programs and packages. The CellchatDB.human was obtained through https://github.com/sqjin/CellChat. Additional information required to reanalyze the data reported in this paper is available from the lead contact upon request.

Research involving human participants, their data, or biological material

Policy information about studies with human participants or human data. See also policy information about sex, gender (identity/presentation), and sexual orientation and race, ethnicity and racism.

Reporting on sex and gender	The donor included in our study were male.
Reporting on race, ethnicity, or other socially relevant groupings	This is not applicable to our study.
Population characteristics	35 human testicular tissues form deceased individuals who consented to organ donation for transplantation and research through DonorConnect for scRNA-seq and experimental validation. The samples were obtained under 20-60 years olds. 20s, n=3; 30s, n=4; 40s, n=10; 50s, n=8; 60s, n=10.
Recruitment	All human testicular tissues were donated samples from deceased individuals who consented to organ donation for transplantation and research through DonorConnect.
Ethics oversight	All human testicular tissues were donated samples from deceased individuals who consented to organ donation for transplantation and research through DonorConnect. Research involving identifiable specimens and/or data from deceased individuals is classified as not involving human subjects according to the NIH Investigator Manual for Human Subjects Research; therefore, IRB approval is not applicable.

Note that full information on the approval of the study protocol must also be provided in the manuscript.

Field-specific reporting

Please select the one below that is the best fit for your research. If you are not sure, read the appropriate sections before making your selection.

☒ Life sciences ☐ Behavioural & social sciences ☐ Ecological, evolutionary & environmental sciences

For a reference copy of the document with all sections, see nature.com/documents/nr-reporting-summary-flat.pdf

Life sciences study design

All studies must disclose on these points even when the disclosure is negative.

Sample size	35 human testicular tissues for scRNA-seq. No statistical method were used to predetermine sample size, Sample sizes were chosen based on prior iterature using similar experimenta paradigms. There were at least three replicates of each group of human samples. The exact sample sizes are in dicated in the figure legends.
Data exclusions	Features with fewer than 200 or more than 5,500 features, UMIs with fewer than 800 or more than 17,000 counts, and cells with greater than 25% mitochondrial mapping were filtered out.
Replication	Most of IF staining were conducted in two donors in each age group. Replications of experiments were successful.
Randomization	35 human testes used for sequencing were divided into six groups according to their ages. 20s: 21y, 22y, 22y; 30s: 30y, 32y, 33y, 38y; 40s: 40y,42y,43y, 43y, 43y, 43y, 44y, 44y, 46y, 48y; 50s: 52y, 53y, 55y, 55y, 57y, 57y, 59y, 59y; 60s: 62y, 64y, 66y, 66y, 66y, 66y, 66y, 67y, 67y,69y. Data collection was randomized for all experiments.
Blinding	Data collection and analysis were conducted without blinding to the experimental conditions. Multiple biological replicates and independent experiments to ensure reproducibility.

Reporting for specific materials, systems and methods

We require information from authors about some types of materials, experimental systems and methods used in many studies. Here, indicate whether each material, system or method listed is relevant to your study. If you are not sure if a list item applies to your research, read the appropriate section before selecting a response.

Materials & experimental systems

n/a	Involved in the study
☐	☒ Antibodies
☒	☐ Eukaryotic cell lines
☒	☐ Palaeontology and archaeology
☒	☐ Animals and other organisms
☒	☐ Clinical data
☒	☐ Dual use research of concern
☒	☐ Plants

Methods

n/a	Involved in the study
☒	☐ ChIP-seq
☒	☐ Flow cytometry
☒	☐ MRI-based neuroimaging

Antibodies

Antibodies used

The following primary antibodies were used for immunofluorescence:
UTF1 Monoclonal Antibody (MFCDA84) (invitrogen, mouse, 1:1000, Cat#14984982)
cKIT (goat, R&D systems,1:1000, Cat#AF332)
Collagen I (rabbit,Abcam, 1:1000, Cat #ab34710)
The following secondary antibodies were used for immunofluorescence:
Donkey anti-mouse AlexaFlour 488 (Invitrogen, Cat#A21202)
Donkey anti-goat AlexaFlour 594 (Invitrogen, Cat#A11508)
Donkey anti-mouse AlexaFlour 488 (Invitrogen, Cat#A21202)

Validation

All of these antibodies were optimized and used previously in the lab and were used in multiple published from our lab such as:
Nie X, Munyoki SK, Sukhwani M, Schmid N, Missel A, Emery BR, DonorConnect, Stukenborg JB, Mayerhofer A, Orwig KE, Aston KI, Hotaling JM, Cairns BR, Guo J. Single-cell analysis of human testis aging and correlation with elevated body mass index. Dev Cell. 2022 May 9;57(9):1160-1176.e5. doi: 10.1016/j.devcel.2022.04.004. Epub 2022 May 2. PMID: 35504286; PMCID: PMC9090997.
Guo J, Nie X, Giebler M, Mlcochova H, Wang Y, Grow EJ; DonorConnect; Kim R, Tharmalingam M, Matilionyte G, Lindskog C, Carrell DT, Mitchell RT, Goriely A, Hotaling JM, Cairns BR. The Dynamic Transcriptional Cell Atlas of Testis Development during Human Puberty. Cell Stem Cell. 2020 Feb 6;26(2):262-276.e4. doi: 10.1016/j.stem.2019.12.005. Epub 2020 Jan 9. PMID: 31928944; PMCID: PMC7298616.
Guo J, Grow EJ, Yi C, Mlcochova H, Maher GJ, Lindskog C, Murphy PJ, Wike CL, Carrell DT, Goriely A, Hotaling JM, Cairns BR. Chromatin and Single-Cell RNA-Seq Profiling Reveal Dynamic Signaling and Metabolic Transitions during Human Spermatogonial Stem Cell Development. Cell Stem Cell. 2017 Oct 5;21(4):533-546.e6. doi: 10.1016/j.stem.2017.09.003. PMID: 28985528; PMCID: PMC5832720.
The source of antibodies were listed as follows:
UTF1 Monoclonal Antibody (MFCDA84): https://www.thermofisher.cn/cn/zh/antibody/product/UTF1-Antibody-clone-MFCDA84-Monoclonal/14-9849-82
cKIT: https://www.rndsystems.com/cn/products/human-cd117-c-kit-antibody_af332
Collagen I : https://www.abcam.cn/products/primary-antibodies/collagen-i-antibody-ab34710.html
